# Tailor-made Janus lectin with dual avidity assembles glycoconjugate multilayers and crosslinks protocells†
†Electronic supplementary information (ESI) available: Synthesis procedures of glycopeptides. Sequences of Janus lectins. SDS gels. ESI mass spectra. SPR sensorgrams. QCM-D profile and data molecular models. Control for vesicules binding. See DOI: 10.1039/c8sc02730g


**DOI:** 10.1039/c8sc02730g

**Published:** 2018-08-14

**Authors:** João P. Ribeiro, Sarah Villringer, David Goyard, Liliane Coche-Guerente, Manuela Höferlin, Olivier Renaudet, Winfried Römer, Anne Imberty

**Affiliations:** a Univ. Grenoble Alpes , CNRS , CERMAV , 38000 Grenoble , France . Email: anne.imberty@cermav.cnrs.fr; b Univ. Grenoble Alpes , CNRS , DCM , 38000 Grenoble , France; c Faculty of Biology , Albert-Ludwigs-University Freiburg , Centre for Biological Signalling Studies (BIOSS) , Schänzlestraße 18 , 79104 Freiburg , Germany . Email: winfried.roemer@bioss.uni-freiburg.de

## Abstract

We engineered the first chimeric, bispecific lectin, with two rationally oriented and distinct recognition surfaces. This lectin, coined Janus lectin in allusion to the two-faced roman god, is able to bind independently to both fucosylated and sialylated glycoconjugates. The multivalent presentation of binding sites on each face of the Janus lectin is very efficient, resulting in avidities in the low nanomolar range for both fucosylated and sialylated surfaces. Moreover, novel heterovalent, bifunctional glycoclusters were synthetized that match the topology of the Janus lectin. Based on these tools, we constructed organized and controlled supramolecular architectures by assembling Janus lectin and glycocompound layer-by-layer. Furthermore, the Janus lectin was employed as biomolecular linker to organize protocells made from giant unilamellar vesicles of different nature, to more complex prototissues. In summary, tailor-made Janus lectins open wide possibilities for creating biomimetic matrices or artificial tissues.

## Abstract 2

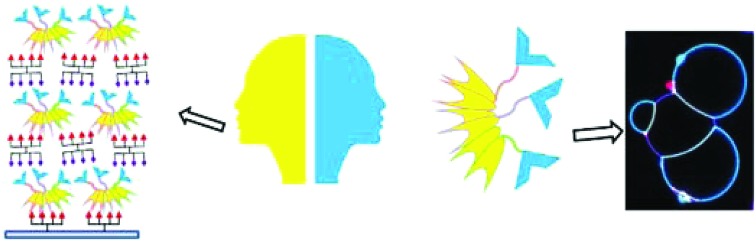
The double-faced Janus lectin, designed by assembling sialic acid and fucose-specific lectin, organize multivalent heteroglyco compounds in mulitlayered material, and glycosylated protocells in prototissues.

## Introduction

Synthetic biology is an emerging discipline for which breathtaking advances have been made in designing and assembling biological components inside modified or artificial cells.^1^–^3^ However, most efforts are devoted to the replication of nucleic acids or the protein machinery, and those for constructing the surface of artificial cells have been limited mainly to biophysical stabilization of the membrane.^4^ The glycocalyx, *i.e.* a meshwork of carbohydrates that forms the interface between the lipid membrane and the environment in all living cells, is generally not taken into account, despite the crucial role of glycoconjugates in cell–cell interactions and tissue development. Insertion of glycolipids in different synthetic membrane systems contributed to the understanding of lipid-mediated endocytosis (reviewed in ref. 5 and 6) of bacterial toxins,^7^ viruses^8^,^9^ or bacteria.^10^ Recently, more complex cholesterol-substituted glycopeptides have been integrated in the creation of protocells, mimicking the glycocalyx present in cell surfaces of living organisms.^11^,^12^

Here, we propose to engineer novel bispecific receptors able to bridge glycoconjugates or protocell surfaces carrying two different types of glycoconjugates for the creation of more complex molecular or nano-objects in an ordered fashion. Such a glycan receptor, coined Janus lectin after the homonymous roman god, can be designed starting from glycan binding proteins (GBPs) that could be either carbohydrate binding modules (CBMs) or lectins. CBMs are small peptide domains, usually associated with carbohydrate-active enzymes.^13^ Their variety of specificity, described in the CAZY database,^14^ and their ability to fold as separated domains make them useful tools in protein fusion technology.^15^ Lectins have fine specificity towards complex oligosaccharides and can bind to glycoconjugates in a multivalent manner resulting in strong avidity that compensates the weaker affinity at each site.^16^

Our goal is to create a novel family of self-assembled proteins with strong dual avidity, able to non-covalently bridge two distinct glycosystems. To this purpose, we designed a chimeric protein by assembling CBM and lectin, generating a supramolecular assembly with two faces, each one with controlled valence and strong avidity for different glycans. The challenge in the construction was (1) to obtain a soluble protein in *Escherichia coli*, (2) to control the oligomerization in order to get multivalent binding and (3) to obtain functional, *i.e.* properly oriented, binding sites for two different glycans. These objects can be used for the creation of functional materials by layering the lectin with new multivalent heteroglyco compounds, and the formation of prototissues by heterogeneous crosslinking of vesicles functionalized with distinct glycomodules.

## Results and discussion

### Design and production of Janus lectin

**Fig. 1:**
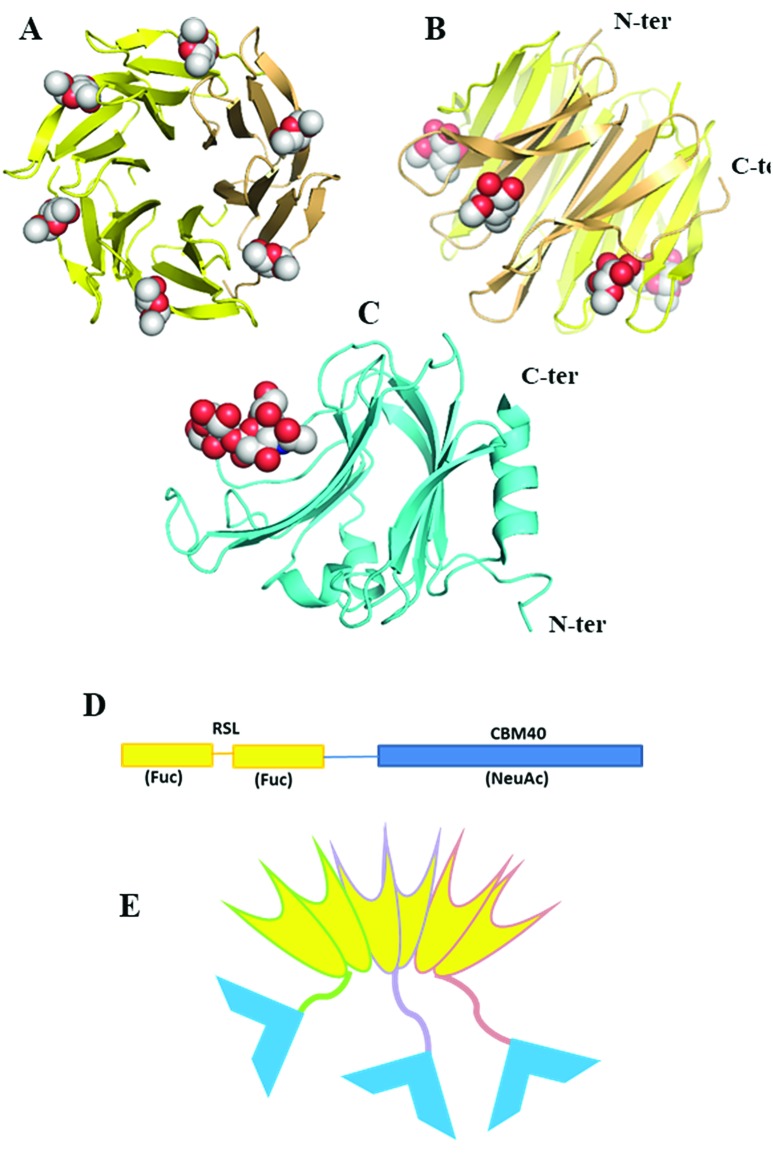
Design and assembling of FS-Janus lectin. (A and B) Two views of crystal structure of RSL-trimer complexed with six fucose ligands (pdb code 2BT9). (C) Crystal structure of CBM40_NanI complexed with 3′-siallylactose (pdb code ; 5FRE). (D) Design of the peptide sequence with two blades of RSL connected to CBM40 through a peptide linker. (E) Schematic representation of the expected FS-Janus lectin presenting six fucose binding sites on the upper face and three sialic acid binding sites on the bottom face.

Since fucose (Fuc) and sialic acid (neuraminic acid: NeuAc) are terminal monosaccharides present on human glycoconjugates, these two epitopes were selected as the two targets of interest. The lectin of *Ralstonia solanacearum* (RSL) adopts a β-propeller trimeric fold with six binding sites oriented on the same side of the protein (Fig. 1A and B) and displays strong affinity for fucose (*K*_D_ = 2 μM)^17^ and remarkable avidity for fucosylated surface (*K*_D_ = 9 nM).^18^ We previously demonstrated that RSL can be engineered, resulting in neoRSL with controlled number of binding sites.^18^,^19^ As for sialic acid, a CBM from the NanI sialidase of *Clostridium perfringens* ATCC13124 (CBM40_NanI) was selected as the protein candidate to be fused to RSL due to the relatively strong affinity for sialylated oligosaccharides (*K*_D_ = 32 μM for 3′-siallyllactose),^20^ the ease of expression, and the accessibility of both C-ter and N-ter extremities for engineering (Fig. 1C). Assembling of both should result in a chimeric and trimeric protein, with two faces able to bind fucose and sialic acid, so noted as FS-Janus lectin.

The gene of the FS-Janus lectin, was designed by fusing the CBM40_NanI sequence to the C-terminus of RSL sequence with including a linker sequence GGGGSGGGGS that should confer flexibility to the supramolecular structure (gene and peptide sequences in ESI Fig. S1†). The production of the recombinant FS-Janus lectin in *E. coli* and purification by affinity chromatography, as previously published for RSL,^17^ resulted in a protein with a molecular weight (MW) of approximately 32 kDa, although most of the production appeared as a 95 kDa protein on SDS PAGE, suggesting the occurrence of the expected trimer, even in denaturing conditions (Fig. S2†). The exact mass determined by ESI-QTof is 31.780 kDa and 95.330 kDa for the trimer (Fig. S3†), which corresponds to the theoretical MW of the chimera construct (31.777 kDa with 9.7 kDa for RSL and 22.0 kDa for CBM40_NanI) assuming depletion of the initial methionine.

### Carbohydrate-binding ability of FS-Janus lectin

**Fig. 2:**
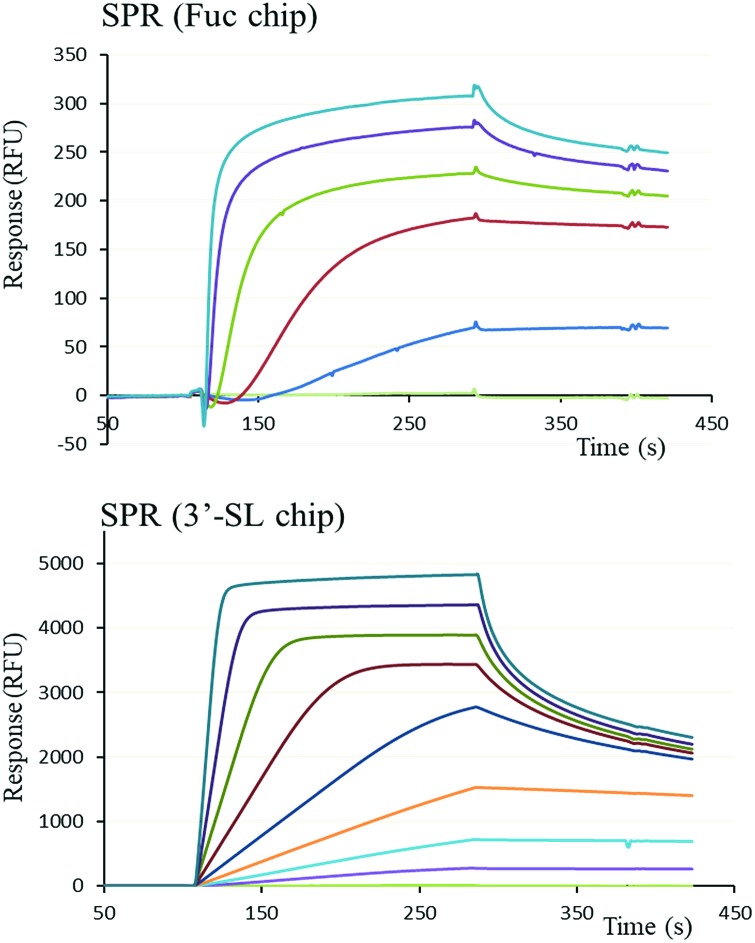
SPR sensorgrams obtained by injection of various concentrations of FS-Janus lectin on a fucose chip (upper) with protein concentrations varying between 26 to 312.5 nM and a 3′-siallyllactose chip (lower) with protein concentrations varying between 9.8 nM to 1.25 μM.

The functional binding activity of FS-Janus lectin was evaluated by surface plasmon resonance (SPR) for testing its avidity towards fucose, 3′-sialyllactose (3′-SL) and 6′-sialyllactose (6′-SL) functionalized chip surfaces. The FS-Janus lectin binds efficiently on both chips (Fig. 2), displaying the fast association and slow dissociation kinetic characteristics for multivalent binding. In both cases, a galactose chip was used for blank channel. The specificity of the binding was verified by regeneration of the chips. After binding of the FS-Janus lectin, the 3′-SL chip could be regenerated by the addition of 100 mM of NeuAc, but not by 1 M fucose. Specificity was also checked for regeneration on the fucose chip that could be performed using fucose, but not NeuAc. Due to complex interaction and multivalency, it was not possible to quantify *k*_on_ and *k*_off_, and apparent affinities were evaluated by steady-state plot analysis (Fig. S4†).

The FS-Janus lectin had a dissociation constant of 78 nM for a fucosylated surface, which is slightly weaker than the one of wild-type RSL (*K*_D_ 9 nM) but 100-fold stronger than what was obtained for a RSL mutant with only one binding site.^19^ An excellent affinity of the FS-Janus protein was also observed for a sialic acid functionalized surface with a *K*_D_ of 68 nM for a 3′-SL chip and 416 nM for 6′-SL chip. The affinity of FS-Janus lectin for 3-linked NeuAc is 200-fold stronger than observed for monomeric CBM40_NanI (*K*_D_ 14.4 μM) for the same surface and 20-fold stronger than the value obtained with a divalent construction of the CBM, and the preference for this linkage is maintained.^20^ The SPR analysis therefore not only confirms that both the sialic-acid specific CBM and the fucose-specific RSL are properly folded with full carbohydrate binding functionality, but moreover, that the resulting orientation of the three CBMs is particularly efficient for binding to sialylated surfaces.

### Assembling FS-Janus lectin in multilayer films

**Fig. 3:**
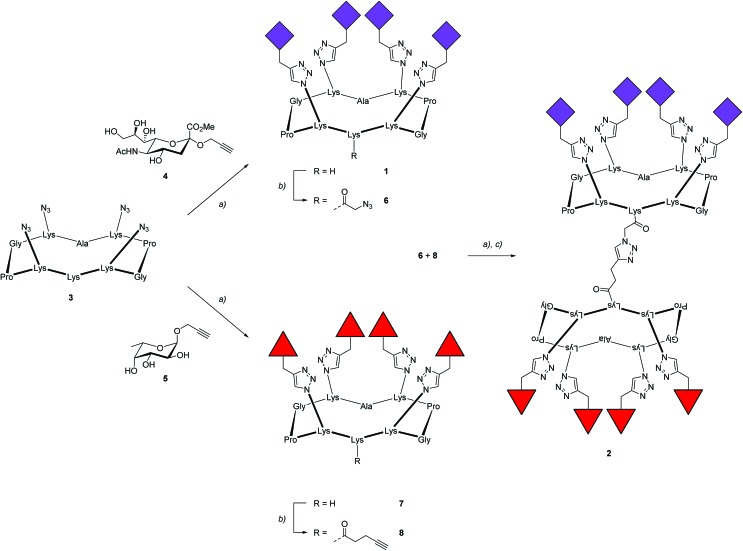
Synthesis of fucosylated and sialylated glycoclusters. Reagents and conditions: (a) CuSO_4_·5H_2_O, tris(3-hydroxypropyltriazolylmethyl)amine (THPTA), PBS buffer (pH 7.5), r.t., 1 h; (b) *N*-succinimidyl azidoacetate or *N*-succinimidyl pentynoate, DIPEA, DMF, r.t., 2 h; (c) LiOH, r.t., 2 h. All amino acids have the L-configuration.

To prove the capacity of the FS-Janus lectin to assemble with different glycosylated molecules and to form supramolecular architectures, we synthesized varieties of glycoclusters built on cyclopeptidic scaffolds (Fig. 3). First, fucosylated cluster **7** and its sialylated analog **1**, were prepared *via* a copper-catalyzed azide alkyne cycloaddition (CuAAC) between propargyl glycosides and cyclopeptidic scaffolds bearing four azide functions.^21^ Next, both tetravalent glycoclusters were further functionalized on their remaining lysine residue with azide (**6**) and propargyl (**8**) linkers, respectively, to allow their assembly by CuAAC and provide heteroglycocluster **2** displaying four fucosides and four sialic acid residues, each presented on two distinct faces of the supramolecule (Fig. 3).

**Fig. 4:**
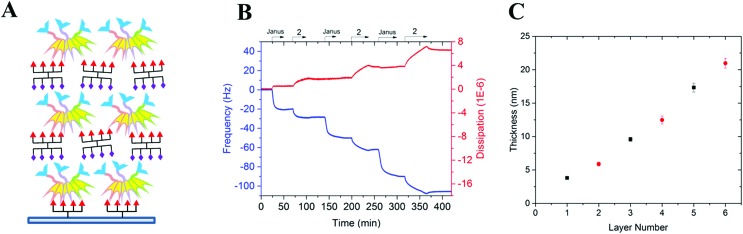
Preparation of a surface-supported multilayer film of alternating layers of FS-Janus lectin and glycoclusters. (A) Representative scheme of the multilayer film: surface-fucosylated cluster **7**/FS-Janus lectin/glycocluster **2**/FS-Janus lectin/… (B) QCM-D profile of the multilayer film construction on fucosylated cluster **7**-functionalized surface. The blue line represents the change in frequency and the red one, the change in dissipation for the 7^th^ overtone. The arrows represent the start and duration of injections; PBS-T was used as running buffer at the starting point for the equilibrium of the signals and then during the rinsing steps between the injections. *T* = 24 °C, flow rate = 10 μL min^–1^. (C) Changes in film thickness calculated by using the viscoelastic model (see Methods) and the experimental values of *f* and *D* measured for the 6 overtones; black square symbols represent FS-Janus lectin, red circle symbols the glycocluster **2**.

Both multivalent compounds were then utilized for building multilayer film through Quartz Crystal Microbalance with Dissipation monitoring technique (QCM-D). Fucosylated cluster **1** was covalently attached on a carboxylic acid-functionalized surface through standard amide coupling (see Fig. S5†) and heteroglycocluster **2** was used in alternation with FS-Janus lectin to build a multilayer film (Fig. 4A). As shown in Fig. 4B, the film growth is characterized by the decrease in frequency accompanying the alternated injections of FS-Janus lectin and the heteroglycocluster **2**.

The film thickness was calculated from the fitting of the QCM-D data (*f* and *D* recorded for six overtones) using a continuum viscoelastic model in the QTM software assuming a highly hydrated film with 1.0 g cm^–3^ density.^22^ The growth is linear up to 20 μm for the 6 layers (Fig. 4C) and the experiments were not pursued further. Simple modeling of both heteroglycocluster **2** and FS-Janus lectins indeed indicated that both have rather similar size, with maximum extension of about 7 nm (Fig. S6†). When analyzing the mass uptake (Fig. S7†), a slightly higher mass is observed for the layer of FS-Janus lectin.

Regarding the changes in dissipation for each layer (Fig. 4B and S8†), we noticed an increase in dissipation during the adsorption of the heteroglycocluster **2** while it kept a constant value in the case of FS-Janus lectin. This observation indicates that the adsorption of the protein on the glycocluster layer leads to a stiffening of the film whereas the heteroglycocluster **2** provides viscous properties to the film. The flexibility of the glycocluster might contribute to the formation of a soft film. This observation has been reinforced by the quantitative measurement of the film softness represented by the acoustic ratio Δ*D*/–Δ*f* (Fig. S9†), which provides information about the mechanical properties of a soft layer. Indeed, the acoustic ratio is much higher in the case of the heteroglycocluster **2** demonstrating that it forms a soft layer compared to FS-Janus lectin that leads to a stiffer layer.

### Cross-linking of glycosylated vesicles by FS-Janus lectin

**Fig. 5:**
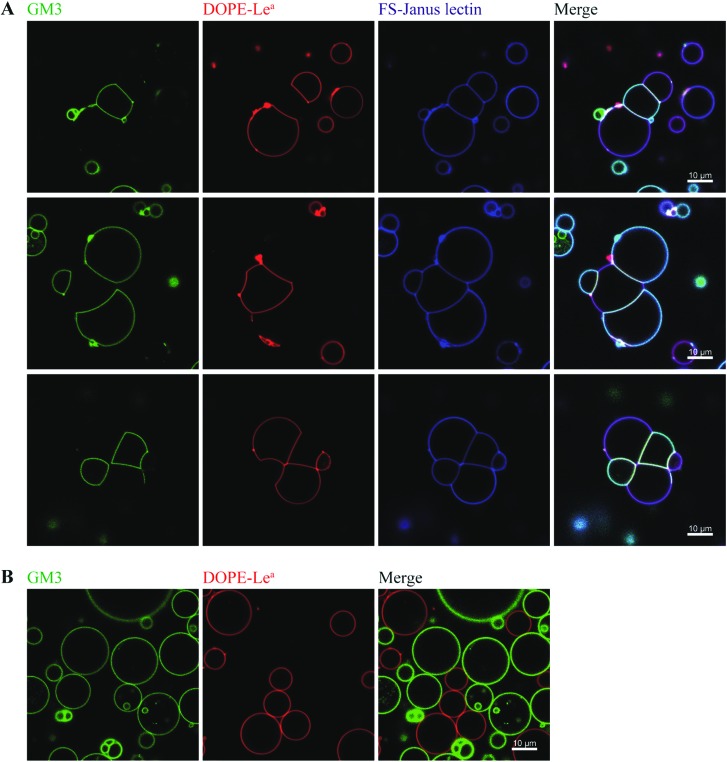
The dual sugar specificity of FS-Janus lectin enables heterogeneous crosslinking of giant vesicles functionalized with distinct glycomodules. (A) Two GUV populations containing 64.5 mol% DOPC, 30 mol% cholesterol and either 5 mol% GM3 and 0.5 mol% BodipyFL-C5-HPC (green), or 5 mol% DOPE-Le^a^ and 0.5 mol% DHPE-TxRed (red) were incubated with 100 nM FS-Janus lectin-AF647 (blue) for 2 h. Due to the dual sugar specificity of FS-Janus lectin, vesicle crosslinking and the formation of elongated interfaces occurred when vesicles of different populations were in close proximity. (B) In order to exclude unspecific interactions, the same GUV populations as in (A) were incubated with PBS, and there was no sign of vesicle adhesion.

By exploiting and extending nature's strategy of using protein–carbohydrate interactions in adhesion events, we investigated the impact of FS-Janus lectin in the organization of protocells into modular organized structures, resembling prototissues. Giant Unilamellar Vesicles (GUVs) are cell-sized spherical lipid bilayer systems considered as protocells, which can be functionalized with different glycoconstructs in order to model a simpler version of the glycocalyx on the cell surface. Two populations of GUVs were prepared by incorporating either the natural glycosphingolipid GM3 with terminal NeuAc or the synthetic DOPE-Lewis^a^ (Le^a^) analog of the native glycolipid, with terminal fucose. GUVs doped with 5 mol% DOPE-Le^a^ were previously demonstrated to bind to RSL.^18^ Addition of the fluorescently labeled FS-Janus lectin (pseudo-colored in blue) to vesicles containing either GM3 (vesicles with green membrane dye) or DOPE-Le^a^ (vesicles with red membrane dye) resulted in lectin binding (turquoise appearing vesicles with GM3 and violet appearing vesicles with DOPE-Le^a^, Fig. 5A, three examples).

Furthermore, progressive crosslinking between the two types of vesicles resulted in the formation of elongated and rather planer interfaces (appearing yellowish). This can be attributed to the dual avidity of FS-Janus lectin, as the addition of the latter to only DOPE-Le^a^ doped vesicles did not show significant crosslinking (Fig. S10A†), which was comparable to the effects observed for native RSL (Fig. S10B†). Yet, FS-Janus lectin was able to crosslink vesicles conjugated with GM3 to some extent (Fig. S10C†). This can most probably be accounted to the flexibility of the CMB40_NanI units on the RSL scaffold, resulting in that the attached CBM can orient away from each other and bind to opposing carbohydrates presented on different vesicles.

Nevertheless, when FS-Janus lectin was applied to both vesicle populations, the crosslinking was much more prominent between vesicles decorated with different glycoconjugates, and the dominating pattern were alternations of GM3-GUVs and DOPE-Le^a^-GUVs (Fig. 5A). In the absence of lectins, the vesicles maintained a spherical shape with minimal contact areas (Fig. 5B).

## Conclusions

Only a very small number of lectins composed of independent domains with different carbohydrate specificities have been identified in nature. One of such cases is the super lectin BC2L-C from *B. cenocepacia*, which recognizes fucose and mannose in binding sites located on different faces of the structure.^23^ Also the human mannose receptor (MR) presents domains with distinct carbohydrate recognition that bind foreign and host ligands.^24^ These peculiar structures could play multiple roles, such as bacterial cross-linking, adhesion to human epithelia or the stimulation of inflammation. We describe here the first engineered lectin presenting different carbohydrate specificities on two faces. Furthermore, the design resulted in high avidity for glycosylated surfaces. The nanomolar avidity for sialylated surfaces is spectacular since classical lectins display low affinity for this important epitope. This property could be of high interest for labeling of epitopes on cell surfaces or for designing drug delivery strategies toward sialylated targets.

The precise topology of our artificial FS-Janus lectin could be matched by unique multivalent compounds that were synthetized for this study. A layer-by-layer approach resulted in novel supramolecular material, composed of alternating lectin and glycoconjugates. To our knowledge, this is the first attempt to build such complexes, based on specific, high affinity interactions. The QCM-D analysis revealed surprising properties: the protein and glycocompound layers have approximately the same thickness, but they reveal very different behavior, the protein layer being stiffer than the carbohydrate one. The design of such novel biomolecular multilayers opens applications in the fields of new functional materials and biosensors. They can also be used for building artificial extracellular matrices for biomimetic applications. The unique property of FS-Janus lectin is to associate distinct glycocompounds or surfaces that expose different glycans. The effect of lectins in cross-linking of glycosylated protocells has been recently demonstrated, and natural lectins with architectures presenting the binding sites on two opposite faces could mimic the formation of cellular junctions.^25^ Our new generation of engineered lectins, now with more complex specificity, will allow for the assembly of protocells, or living cells, with different surface markers, leading to various properties. This is therefore the next step for associating complex cells by protocellular junctions and for building prototissues that are much closer to the ones from living organisms. Because of its precise specificity for glycan structures, and its strong avidity for glycosurfaces, FS-Janus lectin should be suitable for immobilizing living cells on surfaces or attaching them together in a controlled manner. The concept of Janus lectin can be adapted to other glycan epitopes, by changing the CMS attached to the RSL scaffold, opening to an even wider range of applications.

## Materials and methods

### Preparation of FS-Janus lectin

The gene sequence of the FS-Janus lectin, comprising the sequences coding for RSL and CBM40_NanI connected by an intermediate 30 nucleotide sequence, was ordered at Eurofins Genomics after appropriate optimisation. The FS-Janus gene, acquired in a pEX vector, was extracted by digestion with restriction enzymes NdeI and NheI, and subcloned into the vector pET25b(+). The construct was used to transform *E. coli* BL21(DE3) cells. A 5 mL pre-culture of transformed *E. coli* cells was cultured overnight in Luria–Bertani (LB) broth supplemented with 100 μg mL^–1^ ampicillin at 37 °C and with a 180 rpm agitation. The next day, cells were diluted 250-fold and cultured in 1 L of the same medium at 37 °C, until they reached an OD_600_ of 0.6. After 5 min cooling at 4 °C, the culture was moved to an incubator shaker (at 16 °C), where IPTG at a final concentration of 1 mM was added to start protein over-expression. The culture was left incubating for 20 h at 16 °C with a 180 rpm agitation. After incubation, the cell culture was centrifuged at 5000*g* for 15 min at 4 °C. The cell pellet was resuspended in 20 mL of chilled PBS buffer (pH 7.4), and passed through the pressure cell disruptor with a pressure of 1.9 mbar. The lysed cells were collected and centrifuged at 50 000*g* for 30 min. The FS-Janus protein in the cell lysate was purified in a 10 mL agarose d-mannose column (Sigma-Aldrich, M6400), followed by 5 rounds of dialysis against PBS (pH 7.4). The purity was confirmed by SDS-PAGE. A final yield of 7.5 mg of pure Janus protein was obtained per liter of culture.

### Affinity data

SPR experiments were performed using a Biacore X100 biosensor instrument (GE Healthcare) at 25 °C. Biotinylated l-fucose-polyacrilamide (PAA), 3′-SL and 6′-SL (Lectinity) were immobilized on CM5 chips (GE Healthcare) that were pre-coated with streptavidin, following a protocol described in literature.^20^ Biotinylated 3′-SL and 6′-SL were diluted to 1 μg mL^–1^ in HEPES buffer with 0.05% Tween 20 (HBS-T) before being injected in one of the flow cells. Low immobilization levels of 94 and 64 response units were obtained for 3′-SL and 6′-SL respectively. Biotinylated l-fucose-PAA wax mixed with biotinylated D-Gal-PAA (90/10 at 200 μg mL^–1^) before immobilisation to a level of 450 RU. d-Gal-PAA reference surface was always present in flow cell 1, thus allowing for the subtraction of bulk effects and non-specific interactions with streptavidin. The running buffer consisted of the same HBS-T (pH 7.4). The purified protein was injected over the flow cell surface at 30 μL min^–1^ in series of 2-fold dilutions. The dissociation of this analyte was done by passing running buffer during 4–6 min. Surfaces were regenerated with one or two consecutive 30 second injections of either Neu5Ac or Fuc, also at 30 μL min^–1^. The information on the affinity was determined by assuming Langmuir 1:1 binding, using the BIAevaluation software.

### General chemical experimental methods

All chemical reagents were purchased from Aldrich (Saint Quentin Fallavier, France) or Acros (Noisy-Le-Grand, France) and were used without further purification. All protected amino acids and Fmoc-Gly-Sasrin® resin were obtained from Advanced ChemTech Europe (Brussels, Belgium), Bachem Biochimie SARL (Voisins-Les-Bretonneux, France) and France Biochem S.A. (Meudon, France). For peptides and glycopeptides, analytical RP-HPLC was performed on a Waters alliance 2695 separation module, equipped with a Waters 2489 UV/visible detector. Analyses were carried out at 1.23 mL min^–1^ (Interchim UPTISPHERE X-SERIE, C18, 5 μm, 125 × 3.0 mm) with UV monitoring at 214 nm and 250 nm using a linear A–B gradient (buffer A: 0.09% CF_3_CO_2_H in water; buffer B: 0.09% CF_3_CO_2_H in 90% acetonitrile). Preparative HPLC was performed on Waters equipment consisting of a Waters 600 controller and a Waters 2487 Dual Absorbance Detector. Purifications were carried out at 22.0 mL min^–1^ (VP 250 × 21 mm nucleosil 100-7 C18) with UV monitoring at 214 nm and 250 nm using a linear A–B gradient. Progress of reactions was monitored by thin layer chromatography using silica gel 60 F254 pre-coated plates (Merck). Spots were visualised by charring with 10% H_2_SO_4_ in EtOH. Silica gel 60 (0.063–0.2 mm or 70–230 mesh, Merck) was used for column chromatography. ^1^H and ^13^C NMR spectra were recorded on a Bruker Avance III 500 MHz spectrometer and chemical shifts (*δ*) were reported in parts per million (ppm). Spectra were referenced to the residual proton solvent peaks relative to the signal of D_2_O (4.79 ppm for ^1^H). ESI mass spectra of peptides and glycopeptides were measured on an Esquire 3000 spectrometer from Bruker or on an Acquity UPLC/MS system from waters equipped with a SQ2 detector. MALDI-TOF was performed on a AutoFlex Speed Bruker after sample pre-treatment in an OligoR3 microcolumn (Applied Biosystems, USA) using 2,5-dihydroxybenzoic acid matrix (glycopeptides). HRMS analyses were performed on a Waters Xevo® G2-S QTof at Mass Spectrometry facility, PCN-ICMG, Grenoble.

### Quartz crystal microbalance with dissipation monitoring (QCM-D) measurement

QCM-D measurements were performed using Q-Sense E4 instruments (Biolin Scientific) equipped with one to four flow modules. The multilayer films of FS-Janus protein and glycocluster **2** were assembled onto gold-coated crystal sensors (Q-Sense QSX 301). Prior to be used, the quartz crystals were exposed to a UV-ozone treatment for 10 min using UV-ozone cleaner (Jelight Company). A self-assembled monolayer of alkanethiolate containing carboxylic acid end groups was adsorbed onto gold-coated quartz crystals. The sensor was immersed in a 1 mM ethanolic solution of HS-C11-EG6-COOH (Prochimia) for at least 12 h at room temperature. The resulting functionalized gold-coated quartz crystal was then rinsed with ethanol, dried under nitrogen and mounted in the QCM-D flow module. The covalent grafting of the glycocluster **7** was monitored *in situ* in the QCM-D flow module, the surface was first activated by exposing it to a 200 mM EDC and 50 mM NHS aqueous solution, followed by rinsing it with milli-Q water. Afterwards, the measurement chamber was rinsed with PBS before the injection of 100 μM fucosylated cluster **7** in PBS buffer, after reaching the saturation of the QCM-D signals, the measurement chamber was rinsed first with PBS buffer and then with milli-Q water. The unreacted NHS-esters were deactivated by injecting 1 mol L^–1^ ethanolamine at pH 8.5 (see Fig. S5 in the ESI†). After rinsing the flow module with milli-Q water, the measurement chamber was rinsed with PBS buffer containing 0.05% of Tween (PBS-T), PBS-T buffer was used as running buffer for the following experiment. The construction of the multilayer film was performed on the resulting fucosylated cluster **7**-functionalized surface by the alternated exposure of 100 nM solution of FS-Janus lectin in PBS-T, and 10 μM heteroglycocluster **2** in PBS-T. Experiments were conducted in a continuous flow of buffer with a flow rate of 10 μL min^–1^ by using a peristaltic pump (ISM935C, Ismatec, Switzerland). The temperature of the E4 QCM-D platform and all solutions were stabilized to ensure stable operation at 24 °C. All buffers were previously degassed in order to avoid bubble formation in the fluidic system. All experiments were performed in three replicates.

Besides measurement of bound mass (or thickness of the adsorbed layer), which is provided from changes in the resonance frequency *f* of the sensor crystal, the QCM-D technique also provides structural information of biomolecular films *via* changes in the energy dissipation *D* of the sensor crystal. *f* and *D* were measured at the fundamental resonance frequency (4.95 MHz) as well as at the third, fifth, seventh, ninth, eleventh, and thirteenth overtones (*i* = 3, 5, 7, 9, 11 and 13). Normalized frequency shifts Δ*f* = Δ*f*_*i*_/*i* and dissipation shifts Δ*D* = Δ*D*_*i*_ are presented. In the case of homogeneous, quasi-rigid films, the frequency shifts are proportional to the mass uptake per unit area (mQCM), which can be deduced from these Sauerbrey relationship:^26^

1 *m*_QCM_ = –*C*Δ*f*

where the mass sensitivity *C* is equal to 18 ng cm^–2^ Hz^–1^ at *f*_1_ = 4.95 MHz. It should be kept in mind that as the film is solvated, the acoustic areal mass density of the film is composed of the areal mass densities of the adsorbate, *m*_ads_, and the hydrodynamically coupled solvent, *m*_solvent_:

2 *m*_QCM_ = *m*_ads_ + *m*_solvent_

For very soft films, such as the layer-by-layer assemblies of FS-Janus protein and glycocluster **2**, eqn (2) is not valid. In the present study, the film thickness was determined by fitting the QCM-D data at selected time points Δ*D* and Δ*f* as a function of *i* to a continuum viscoelastic model with the software QTM (D. Johannsmann, Technical University of Clausthal, Germany; ; http://www.pc.tu-clausthal.de/en/research/johannsmann-group/qcm-modelling; option “small load approximation”)^22^ following a procedure previously described.^27^ The thickness values correspond to mean values of the best fit thicknesses of three independent experiments and the confidence intervals have been calculated from these three experiments. In this model, the thickness was extracted from a set of *f* and *D* experimental data for six overtones, considering a highly hydrated film, the film density *ρ* was fixed to 1.0 g cm^–3^.

The acoustic ratio Δ*D*/–Δ*f* was used to calculate the elastic compliance *J*′(*f*) (a measure for film softness) *via*eqn (1),^28^ and thus provides information on the mechanical properties of a soft layer:3
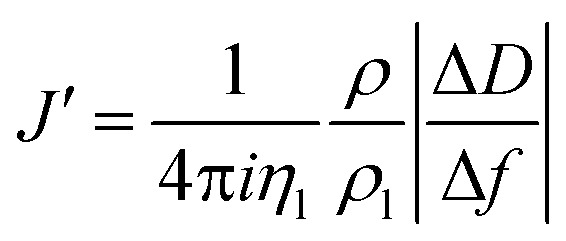
where *η*_l_ = 0.89 mPa s and *ρ*_l_ = 1 g cm^–3^ are the viscosity and the density of the aqueous bulk solution, respectively, and *ρ* is the film density.

### Preparation of GUVs

GUVs were made by the electroformation technique as previously described.^29^ In brief, a total concentration of 0.5 mg mL^–1^ lipids dissolved in chloroform were deposited on indium tin oxid-covered glass slides and dried under vacuum. Two slides were assembled to a chamber, filled with 265 mOsm L^–1^ sucrose, and an alternating electrical field with a field strength of 1 V mm^–1^ was applied for 3 h at room temperature. GUVs were observed in home-built chambers as described.^29^ Images were collected with a confocal fluorescence microscope (Nikon Eclipse Ti-E inverted microscope equipped with a Nikon A1R confocal laser scanning system, 60× oil immersion objective, NA = 1.49, 4 laser lines: 405 nm, 488 nm, 561 nm, 640 nm). The software NIS-elements (Nikon) was used for image acquisition and analysis.

## Conflicts of interest

There are no conflicts to declare.

## Supplementary Material

Supplementary information
