# Supplementary material for: Tailor-made Janus lectin with dual avidity assembles glycoconjugate multilayers and crosslinks protocells
Source: Chem Sci. 2018 Aug 14;9(39):7634–41. doi: 10.1039/c8sc02730g (PMC6182566; doi:10.1039/c8sc02730g)
Supplement: Supplementary file 1 [file SC-009-C8SC02730G-s001.pdf]

Electronic Supplementary Material (ESI) for Chemical Science.
This journal is © The Royal Society of Chemistry 2018

Tailor-made Janus lectin with dual avidity assembles glycoconjugate multilayers and crosslinks protocells

João P. Ribeiro, Sarah Villringer, David Goyard, Liliane Coche-Guerente, Manuela Höferlin, Olivier Renaudet, Winfried Römer, and Anne Imberty

Supporting information

A

ATGAGCAGCGTTCAGACCGCGGCGACCAGCTGGGGCACCGTTCCGAGCATCCGTGTTTACACCGCGAATAATGGCAAAAT
CACCGAGCGTTGCTGGGACGGTAAAGGCTGGTACACCGGTGCGTTCAACGAGCCGGGCGATAACGTGAGCGTTACCAGC
TGGCTGGTTGGTAGCGCGATCCACATTCGTGTTTATGCGAGCACCGGCACCACCACCACCGAATGGTGCTGGGACGGTAA
CGGCTGGACCAAGGGTGCGTATACCGCGACCAATGGTGGCGGTGGCAGCGGTGGCGGTGGCAGCCTGAGCAGCCTGGG
CGAGTATAAGGATATCAACCTGGAAAGCAGCAACGCGAGCAACATTACCTACGACCTGGAGAAGTATAAAAACCTGGATG
AAGGTACCATCGTGGTTCGTTTCAACAGCAAGGACAGCAAAATCCAGAGCCTGCTGGGTATTAGCAACAGCAAGACCAAA
AACGGCTACTTCAACTTTTATGTGACCAACAGCCGTGTTGGTTTTGAGCTGCGTAACCAGAAAAACGAGGGTAACACCCAA
AACGGCACCGAGAACCTGGTGCACATGTACAAGGATGTTGCGCTGAACGACGGTGATAACACCGTGGCGCTGAAAATCG
AAAAGAACAAAGGTTATAAGCTGTTTCTGAACGGCAAGATGATTAAAGAGGTTAAGGACACCAACACCAAGTTCCTGAAC
AACATCGAAAACCTGGATAGCGCGTTTATTGGTAAAACCAACCGTTACGGCCAAAGCAACGAGTATAACTTCAAGGGTAA
CATTGGCTTTATGAACATCTACAACGAACCGCTGGGTGATGATTATCTGCTGAGCAAAACCGGCGAAACCAAGTAA

B

MSSVQTAATSWGTVPSIRVYTANNGKITERCWDGKGWYTGAFNEPGDNVSVTSWLVGSAIHIRVYASTGTTTTE
WCWDGNGWTKGAYTATNGGGGSGGGGSLSSLGEYKDINLESSNASNITYDLEKYKNLDEGTIVVRFNSKDSKIQSL
LGISNSKTKNGYFNFYVTNSRVGFELRNQKNEGNTQNGTENLVHMYKDVALNDGDNTVALKIEKNKGYKLFLNGK
MIKEVKDTNTKFLNNIENLDSAFIGKTNRYGQSNEYNFKGNIGFMNIYNEPLGDDYLLSKTGETK

Figure S1. A. Design of optimized gene for expression of FSJanus lectin. B. Resulting amino acid sequence with color coding for RSL domain (yellow), linker (grey) and CBM-NanI (blue).

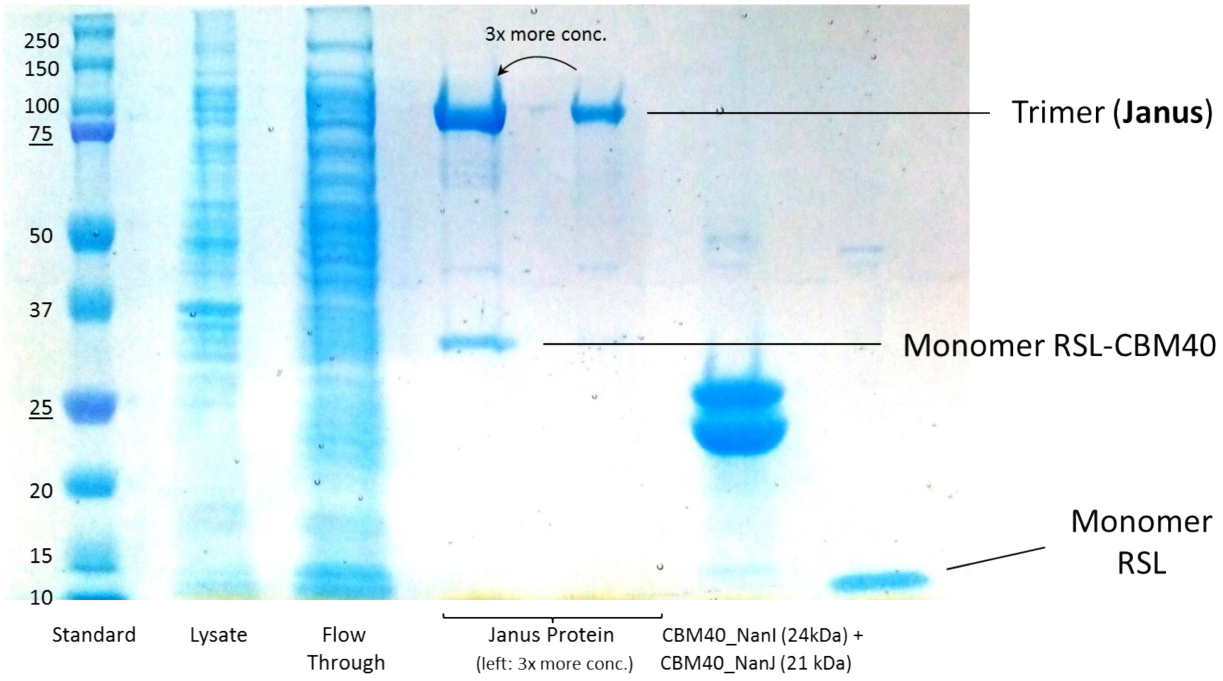

Figure S2. Denaturing SDS gel. 15% acrylamide before and after purification of FS-Janus lectin, in comparison with RSL and CBM40.

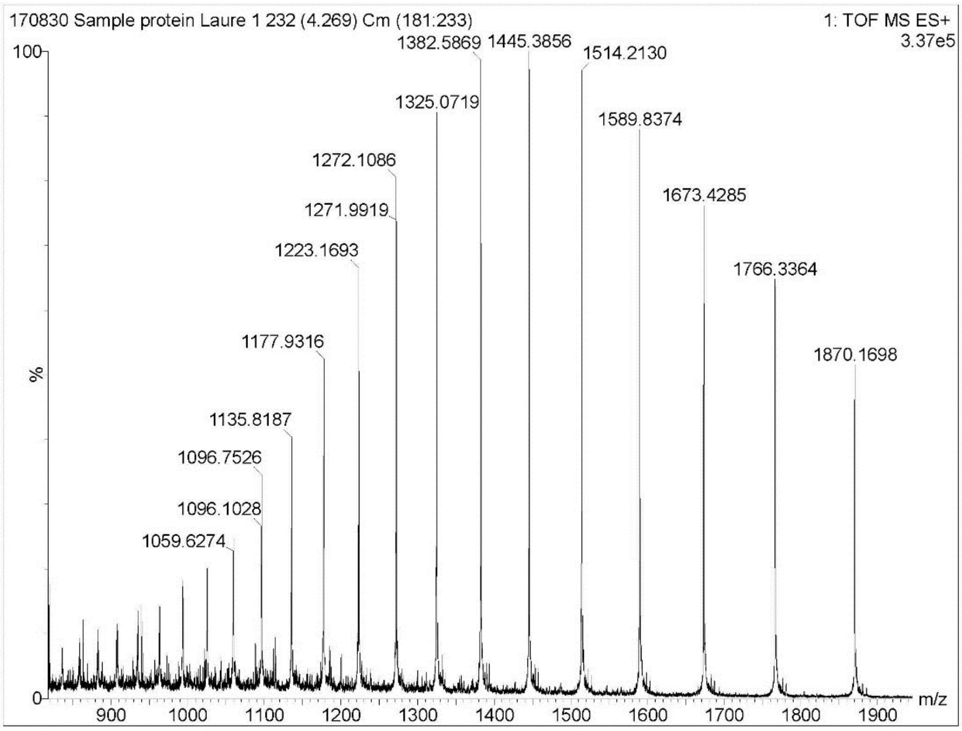

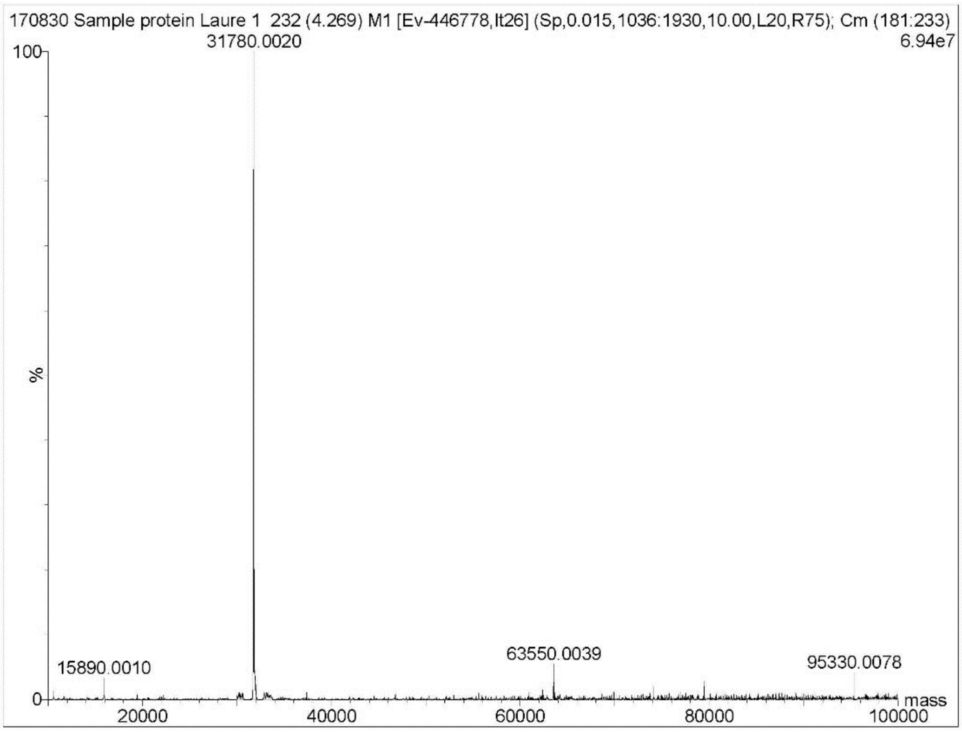

Figure S3. ESI mass spectra of FS-Janus lectin. Original m/z spectrum (top) and MaxEnt1-processed spectrum on a true molecular mass scale (bottom).

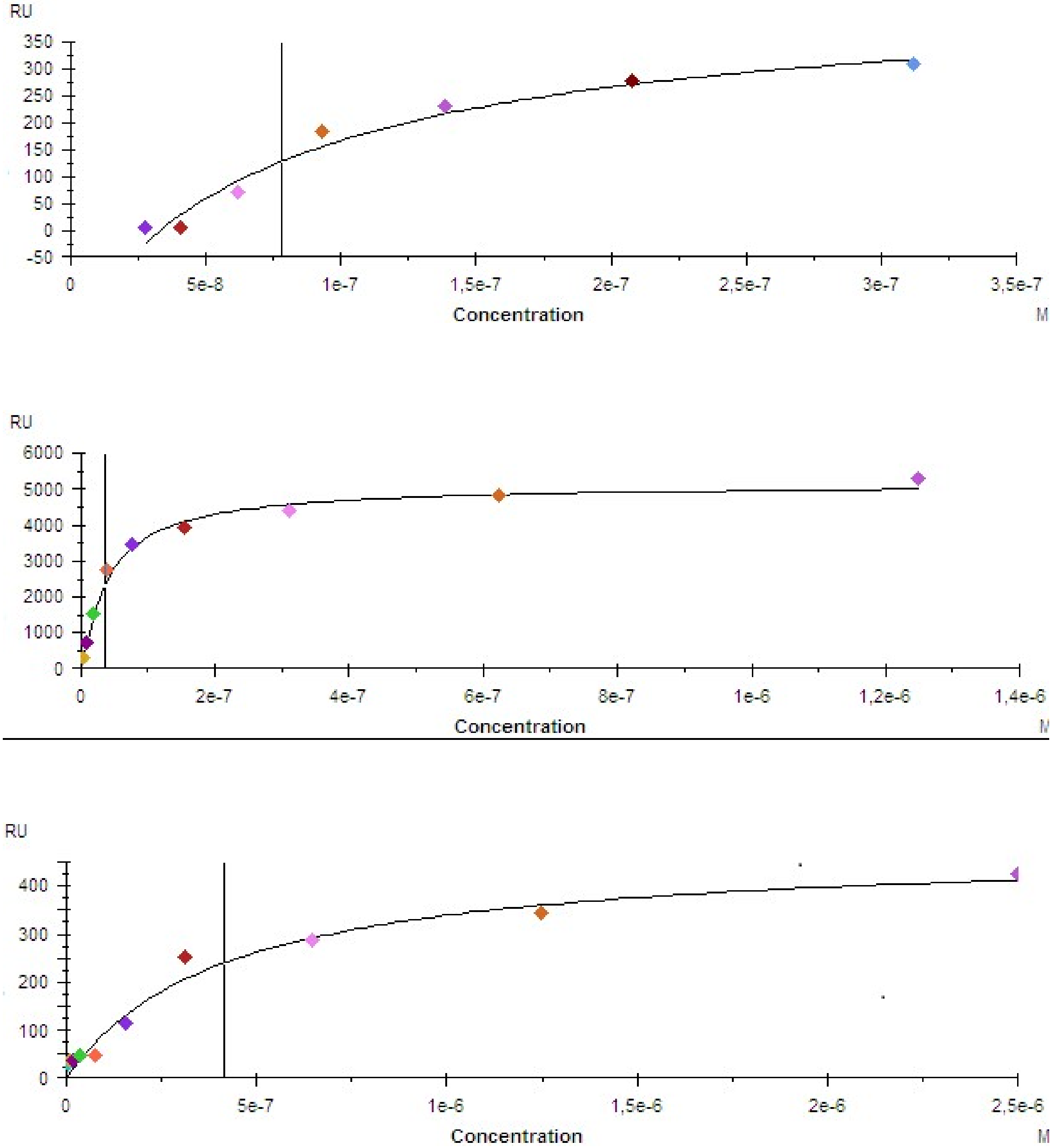

Figure S4. Steady state analysis of SPR data for FS-Janus lectin on fucose chip (top), 3'sialyllactose chip (middle) and 6'sialyllactose chip (bottom).

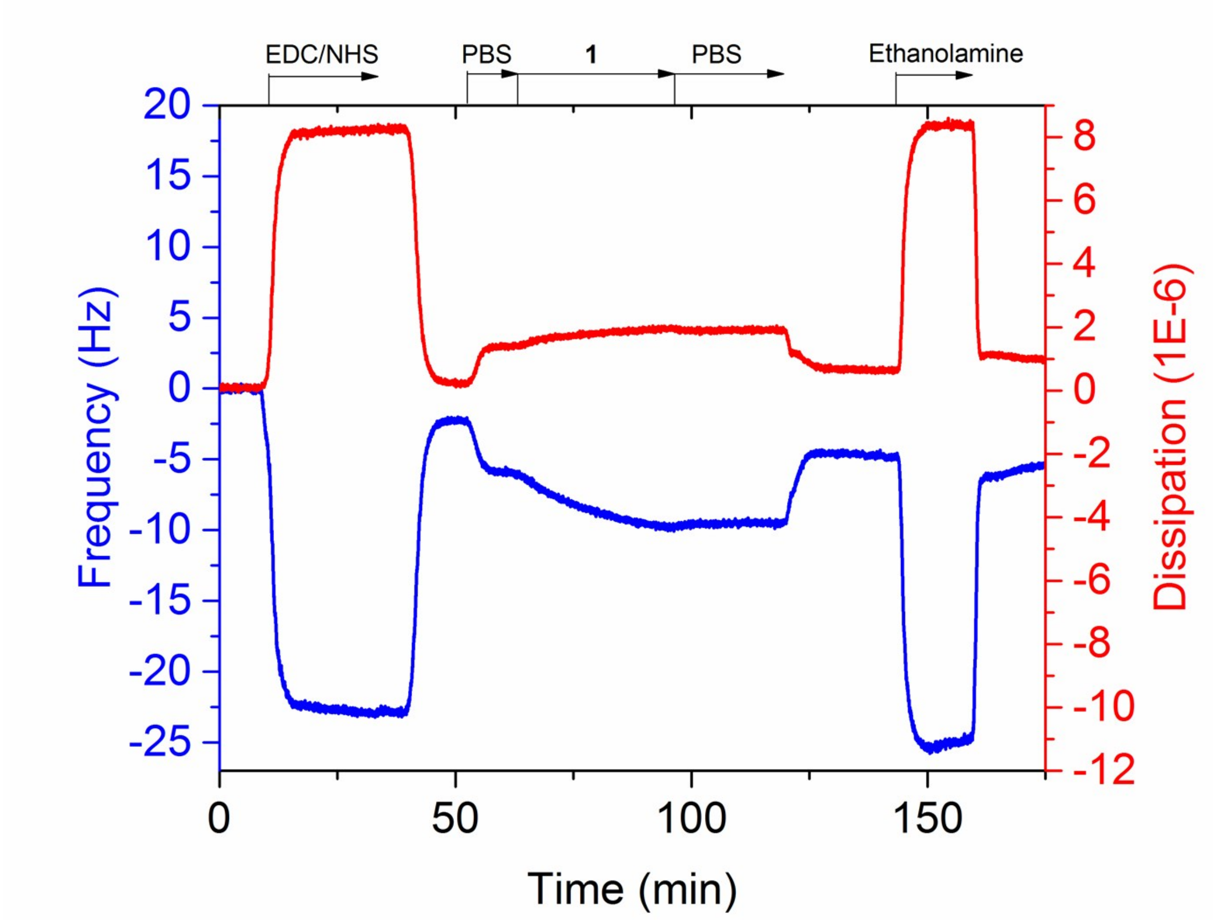

Figure S5. QCM-D profile recorded during the covalent grafting of Fucosylated cluster **1** on carboxylic acid-functionalized gold quartz surface. The blue line represents the change in frequency and the red one, the change in dissipation for the 7th overtone. The arrows represent the start and duration of injections, milliQ-water was used first for the equilibrium of the signals before the injections of EDC/NHS and in between each injections, T = 24°C, flow rate = 10 μL min^{-1}.

As the add-layer of Fucosylated cluster **1** is rigid (the change in dissipation is very low), Sauerbrey equation (1) can be applied to calculate the mass uptake: 64.55 ± 2.71 ng cm^{-2}.

$$m_{QCM} = - C.\Delta f \qquad (1)$$

where the mass sensitivity, C, is equal to 18 ng cm^{-2} Hz^{-1} at f_1 = 4.95 MHz.

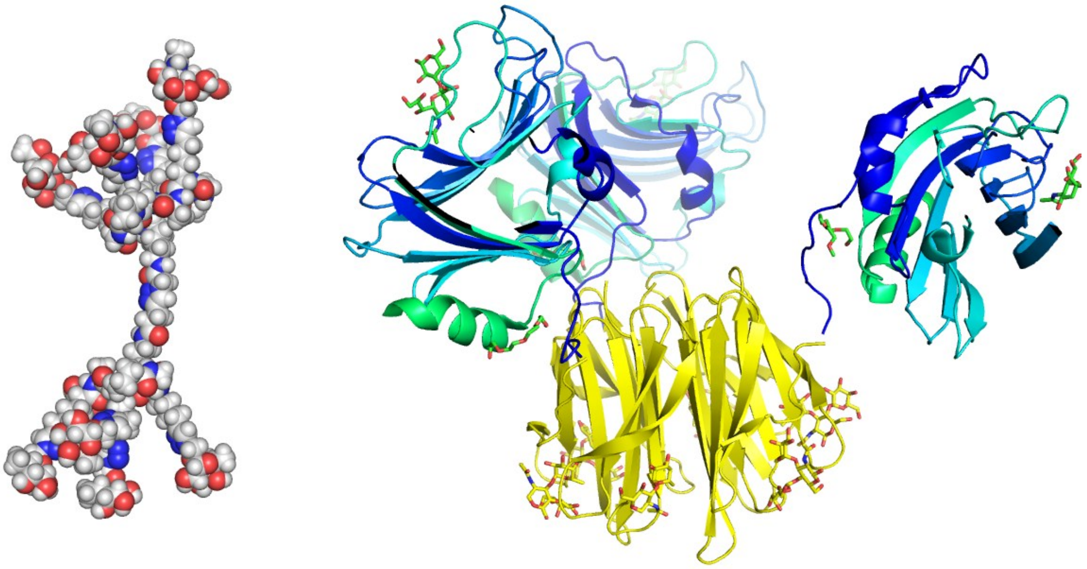

Figure S6. Model of heteroglycocluster 2 in extended conformation and of FS-Janus lectin built by juxtaposition of three CBM40_NanI n-ter to the three RSL C-ter extremities (no energy calculations). The two models are built at the same scale for purpose of size comparison.

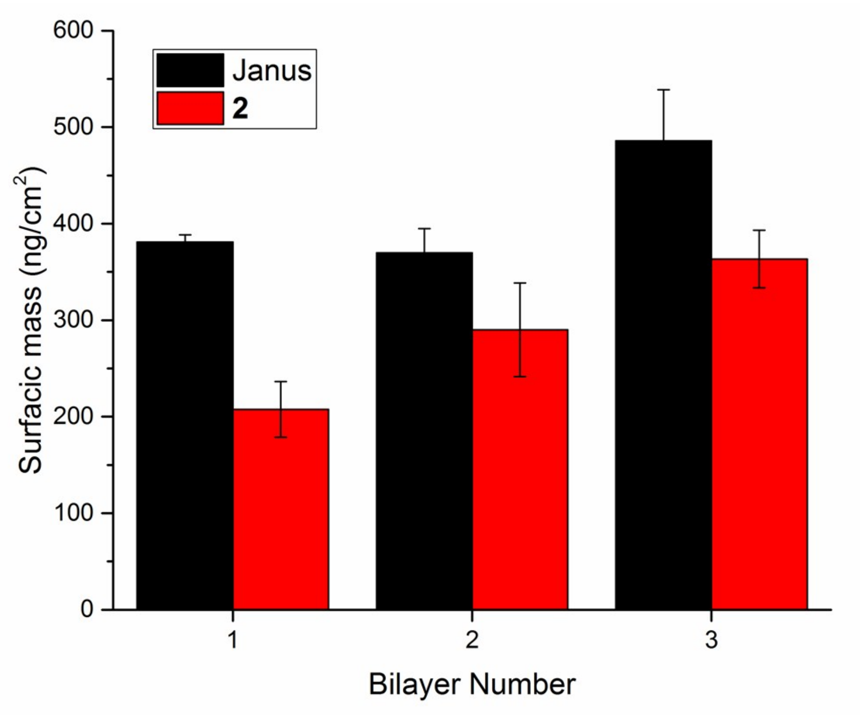

Figure S7. The histograms show the evolution of the acoustic mass of each layer (FS-Janus lectin in black and Heteroglycocluster 2 in red) calculated by using the viscoelastic modeling with the software QTM (D. Johannsmann, Technical University of Clausthal, Germany; http://www.pc.tu-clausthal.de/en/research/johannsmann-group/qcm-modelling; option "small load approximation"). The numbers in X-axis correspond to the number of the bilayer (FS-Janus lectin /Glycocluster 2)$_n$.

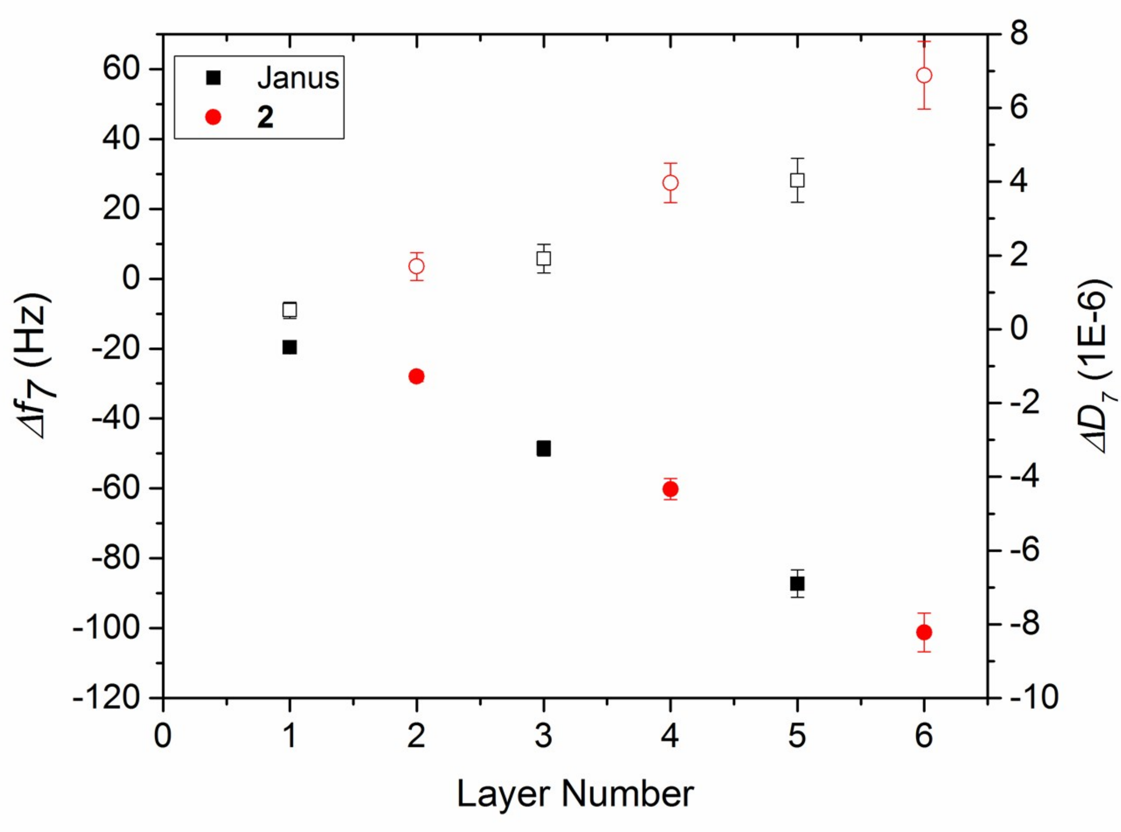

Figure S8. Shifts in frequency (filled symbols) and dissipation (open symbols) recorded during the construction of the multilayer film (FS-Janus lectin (black square)/ Heteroglycocluster **2** (red circle) on Fucosylated cluster **1**-functionalized surface. The present data resulted from the mean values of three independent measurements and the errors bars represent the confidence intervals.

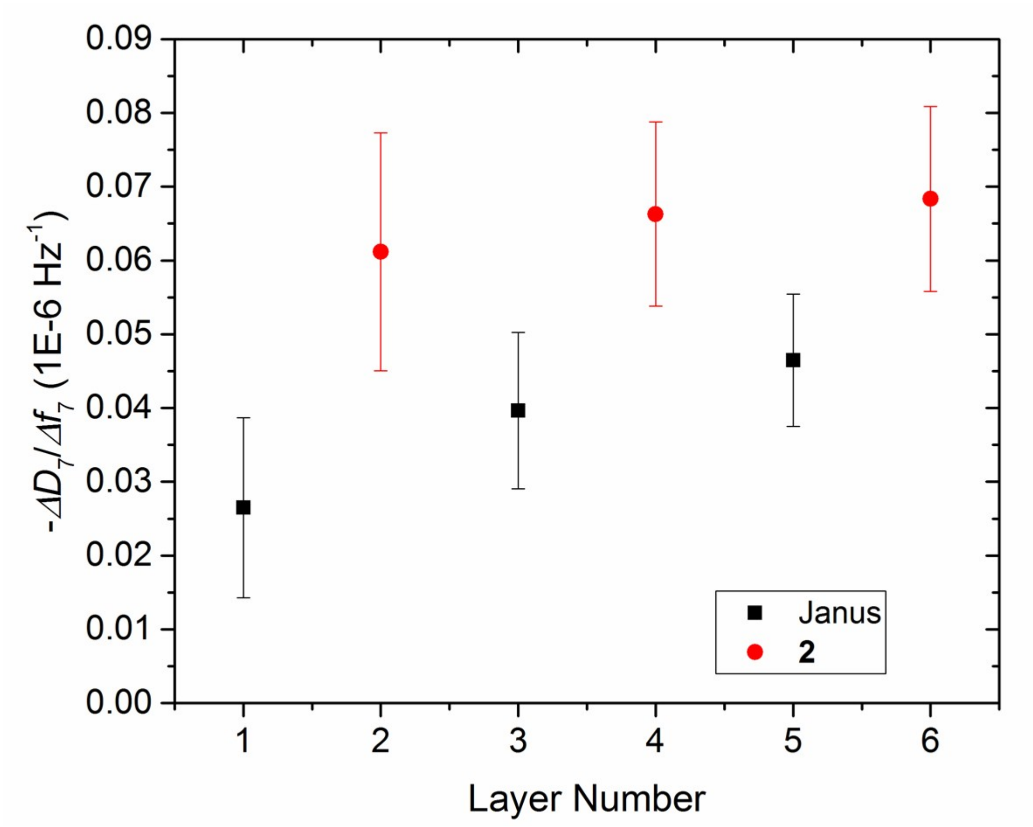

Figure S9. Plot of the acoustic ratio $\Delta D/-\Delta f$ (7th overtone) for FS-Janus lectin layer (black square) and Heteroglycocluster **2** layer (red circle) as a function of the layer number. The data represent the mean values of three independent measurements and the error bars the confidence intervals.

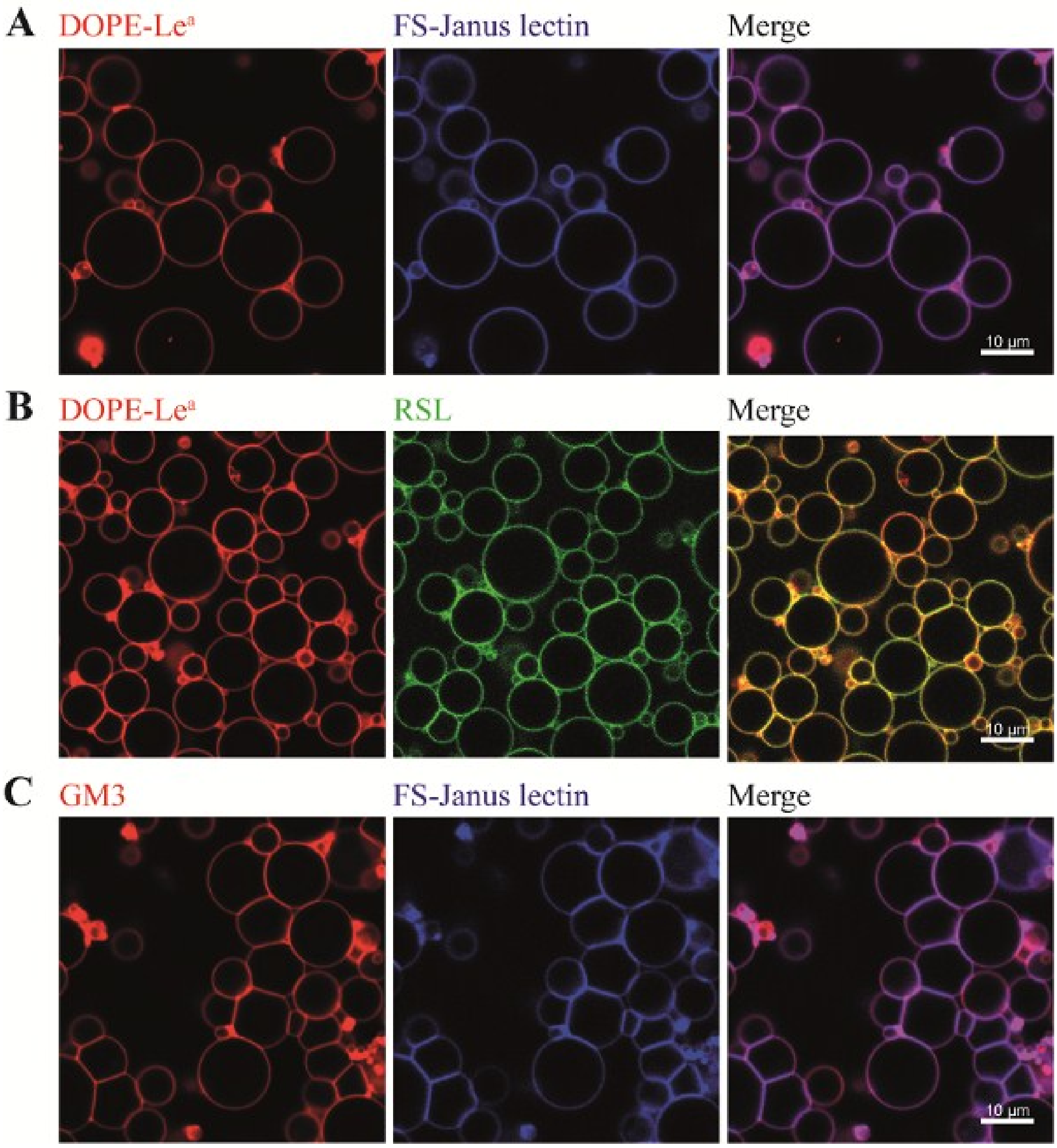

Figure S10. Homogenous lectin binding or crosslinking of FS-Janus lectin or RSL. (A+B) Vesicles were composed of 64.5 mol% DOPC, 30 mol% cholesterol, 5 mol% DOPE-Le^a and 0.5 mol% DHPE-TxRed (red). (A) Incubation with 100 nM FS-Janus lectin-AF647 (blue) for 2 h resulted in binding, but not the formation of interfaces as there were no vesicles functionalized with GM3. Accordingly, the RSL domain of FS-Janus lectin should not be able to bind to two vesicles in parallel. (B) In relation to (A) 100 nM RSL-AF488 (green), which represents one domain of FS-Janus lectin, did bind but not crosslink, as the lectin does not possess opposing binding sites. (C) Vesicles containing 64.5 mol% DOPC, 30 mol% cholesterol, 5 mol% GM3 and 0.5 mol% DHPE-TxRed (red) were incubated with 100 nM FS-Janus lectin-AF647 for 2 h. Elongated interfaces, which altered the spherical shape of the interfaces, indicate that the CBM40 domain of FS-Janus lectin consisting of three CBM40 monomers is able to bind to more than one vesicle in parallel. This effect can most probably be attributed to the flexibility of the linker connecting each of the individual CBM40 monomers to each monomer of the RSL trimer.

Synthetic procedures and compounds' characterization

Cyclopeptide **3** (102 mg, 91 μmol) was obtained adapting the already described protocol from Renaudet *et al.* in *J. Carbohydr. Chem.* **2011**, *30*, 458–468. HRMS (ESI$^+$-TOF) *m/z*: calcd for $C_{47}H_{78}N_{23}O_{10}$ [M+H]$^+$: 1124.6302, found 1124.628; RP-HPLC: R_t = 8.61 min (C_{18}, λ = 214 nm 5-80% B in 15 min). 2-propynyl α-L-fucopyranoside **4** was synthesized in three steps from L-fucopyranose following an already published protocol. (Bergeron-Brlek *et al.*, *Carbohydr. Res.*, **2011**, *346*, 1479-1489) Methyl (2-propynyl 5-acetamido-3,5-dideoxy-D-glycero-α-D-galacto-2-nonulopyranosid)onate **5** was synthesized in four steps from *N*-acetylneuraminic acid following an already published protocol. (Ribeiro *et al., Biochem. J.*, **2016**, *473*, 2109-2118).

Fucosylated tetravalent glycocluster (1)

A solution of $CuSO_4{\cdot}5H_2O$ (5.3 mg, 21.4 μmol, 0.6 eq.), THPTA (19 mg, 42.7 μmol, 1.2 eq.) and sodium ascorbate (21 mg, 107 μmol, 3 eq.) in PBS buffer (500 μL, pH 7.5) was added to a solution of scaffold **3** (40 mg, 35.6 μmol, 1eq.) and propargyl fucoside **4** (77 mg, 214 μmol, 6 eq.) in 500 μL of a 1:1 mixture of DMF/PBS buffer (pH 7.5). The mixture was degassed under argon and stirred at room temperature for 1 h after which UPLC analysis showed complete coupling. Chelex resin was added to the reaction mixture which was then purified by semipreparative RP-HPLC (5-40% solvent B in 15 min) to afford compound **1** (77.6 mg, 40.1 μmol, 85%) as a white fluffy solid after lyophilization. HRMS (ESI$^+$-TOF): *m/z* calcd for $C_{83}H_{134}N_{23}O_{30}$ [M+H]$^+$ 1932.9667; found 1932.9701; RP-HPLC: R_t = 6.60 min (C18, λ = 214 nm, 5-40% solvent B in 15 min).

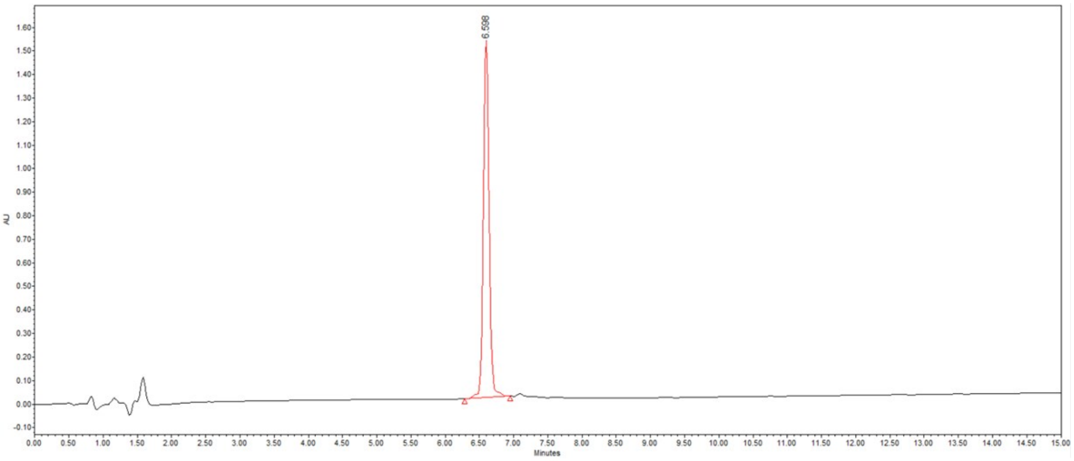

Figure S11. Analytical HPLC spectrum of compound **1**.

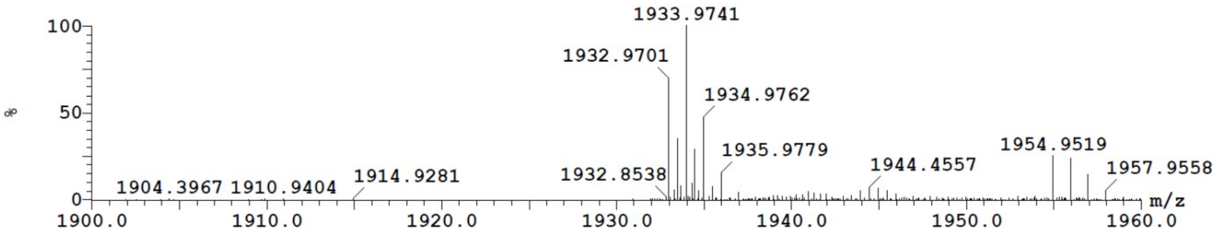

Mass	PPM	i-FIT (norm)	Formula	Calc. Mass
1932.9701	1.8	1.4	C83 H134 N23 O30	1932.9667

Figure S12. HRMS spectrum of compound **1**.

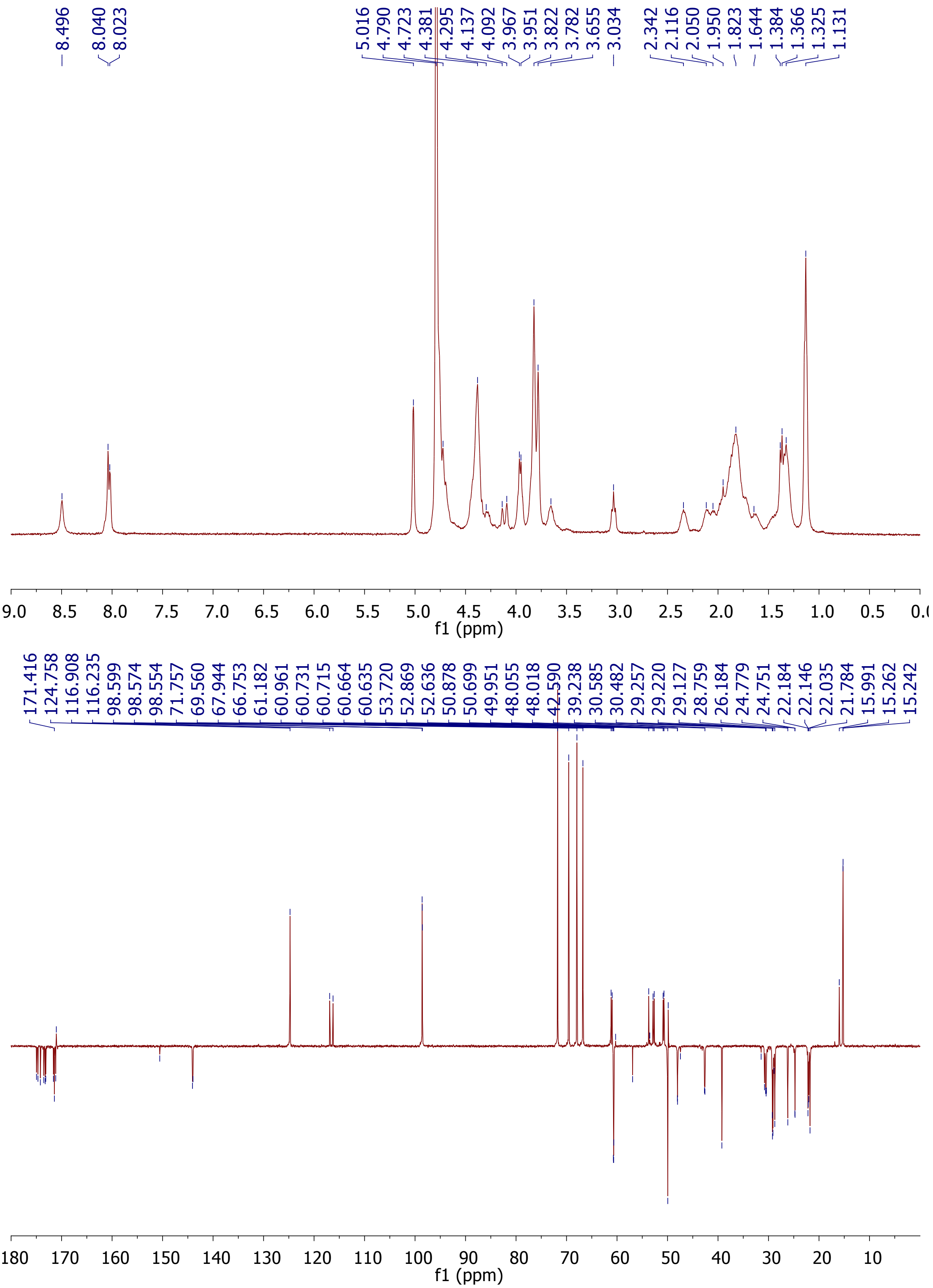

Figure S13. ^{1}H (top) and ^{13}C NMR (bottom) spectra of compound **1** in D_2O at 500 and 125 MHz respectively.

Propargyl-functionalized fucosylated tetravalent glycocluster (6)

To a solution of **1** (35 mg, 18 µmol, 1 eq.) in dry DMF (500 µL) were added diisopropylethylamine (9.4 µL, 54 µmol, 3 eq.) and *N*-succinimidyl pentynoate (5.3 mg, 27 µmol, 1.5 eq.). The mixture was stirred at room temperature (r.t.) for 1 h after which UPLC analysis showed complete conversion. H_2O (3 mL) was then added to the mixture which was purified by semipreparative RP-HPLC (5-40% solvent B in 15 min) to afford compound **6** (31.9 mg, 15.8 µmol, 88%) as a white fluffy solid after lyophilization. HRMS (ESI$^+$-TOF): *m/z* calcd for $C_{88}H_{137}N_{23}NaO_{31}$ [M+Na]$^+$ 2034.9749; found 2034.9777; RP-HPLC: R_t = 7.88 min. (C18, λ = 214 nm, 5-40% solvent B in 15 min).

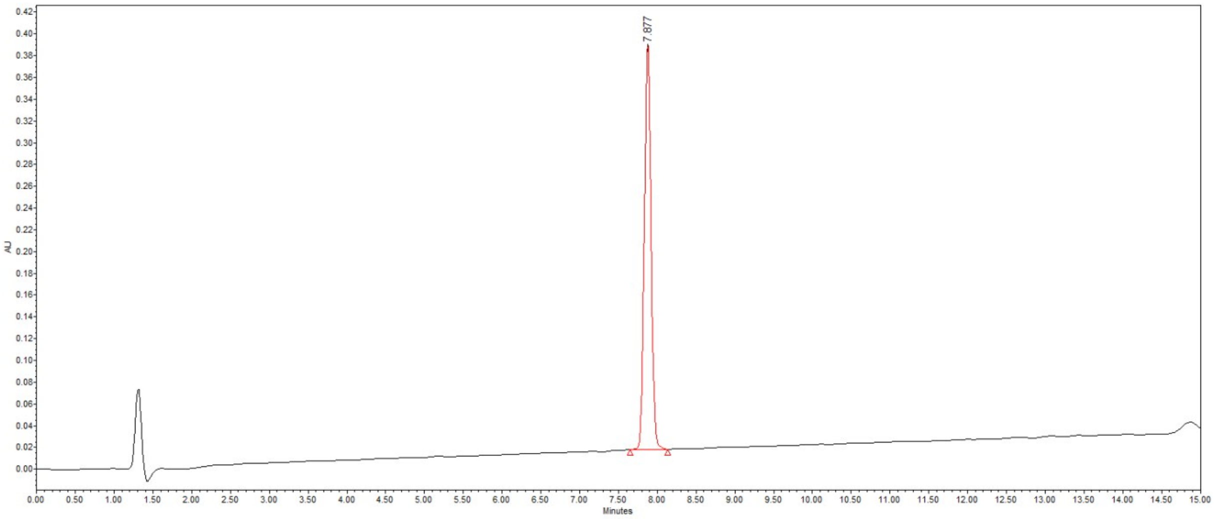

Figure S14. Analytical HPLC spectrum of compound **6**.

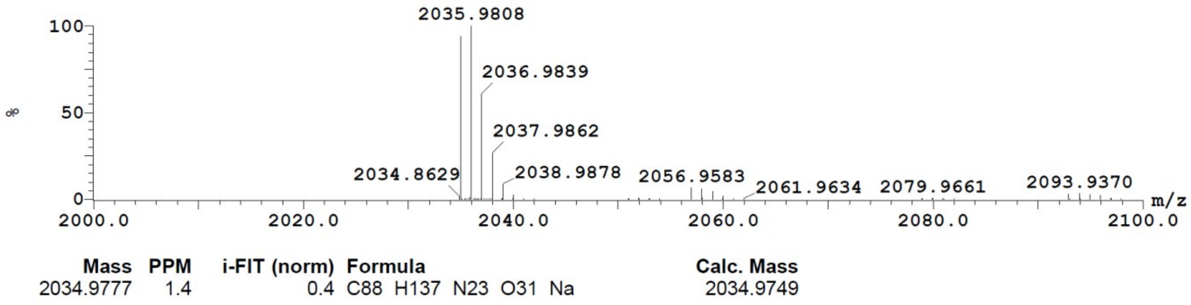

Figure S15. HRMS spectrum of compound **6**.

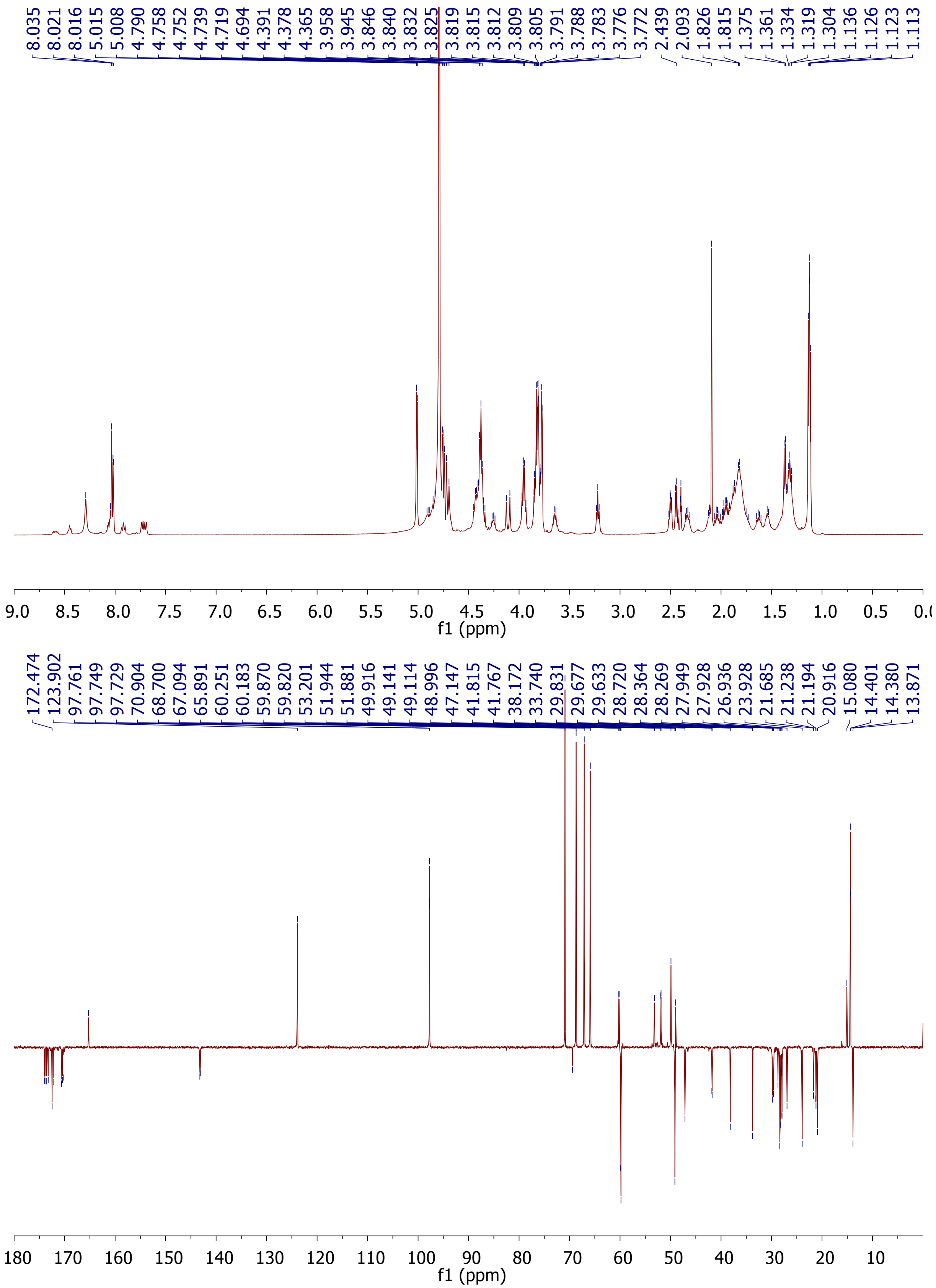

Figure S16. ^{1}H (top) and ^{13}C NMR (bottom) spectra of compound **6** in D_2O at 500 and 125 MHz respectively.

Sialylated tetravalent glycocluster (7)

A solution of $CuSO_4 \cdot 5H_2O$ (5.5 mg, 21.9 µmol, 0.6 eq.), THPTA (19 mg, 43.8 µmol, 1.2 eq.) and sodium ascorbate (22 mg, 109 µmol, 3 eq.) in PBS buffer (500 µL, pH 7.5) was added to a solution of scaffold **3** (41 mg, 36.5 µmol, 1eq.) and propargyl sialoside **5** (79 mg, 219 µmol, 6 eq.) in 500 µL of a 1:1 mixture of DMF/PBS buffer (pH 7.5). The mixture was degassed under argon and stirred at room temperature for 1 h after which UPLC analysis showed complete coupling. Chelex resin was added to the reaction mixture which was then purified by semipreparative RP-HPLC (5-40% solvent B in 15 min.) to afford compound **7** (74.0 mg, 28.8 µmol, 79%) as a white fluffy solid after lyophilization. HRMS (ESI$^+$-TOF): *m/z* calcd for $C_{107}H_{169}N_{27}NaO_{46}$ $[M+Na]^+$ 2591.1613; found 2591.1616; RP-HPLC: R_t = 6.45 min. (C18, λ = 214 nm, 5-40% solvent B in 15 min).

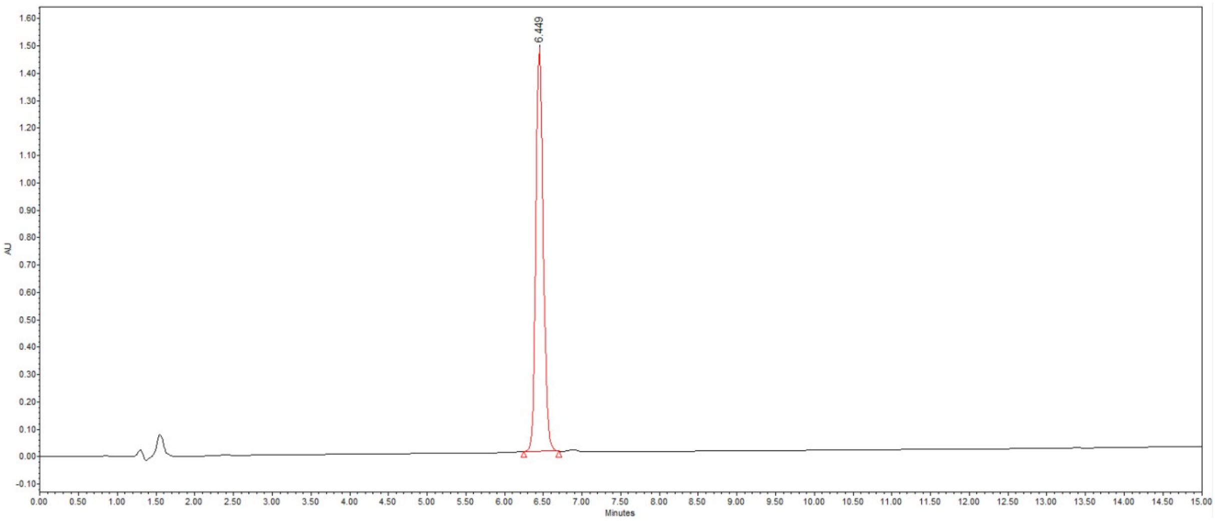

Figure S17. Analytical HPLC spectrum of compound **7**.

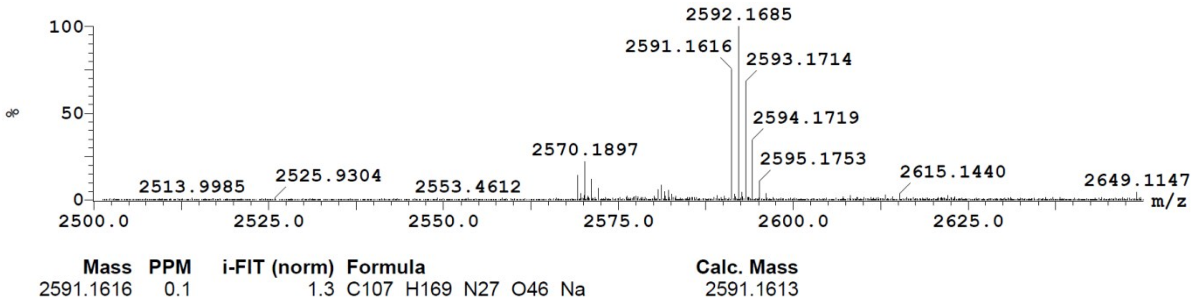

Figure S18. HRMS spectrum of compound **7**.

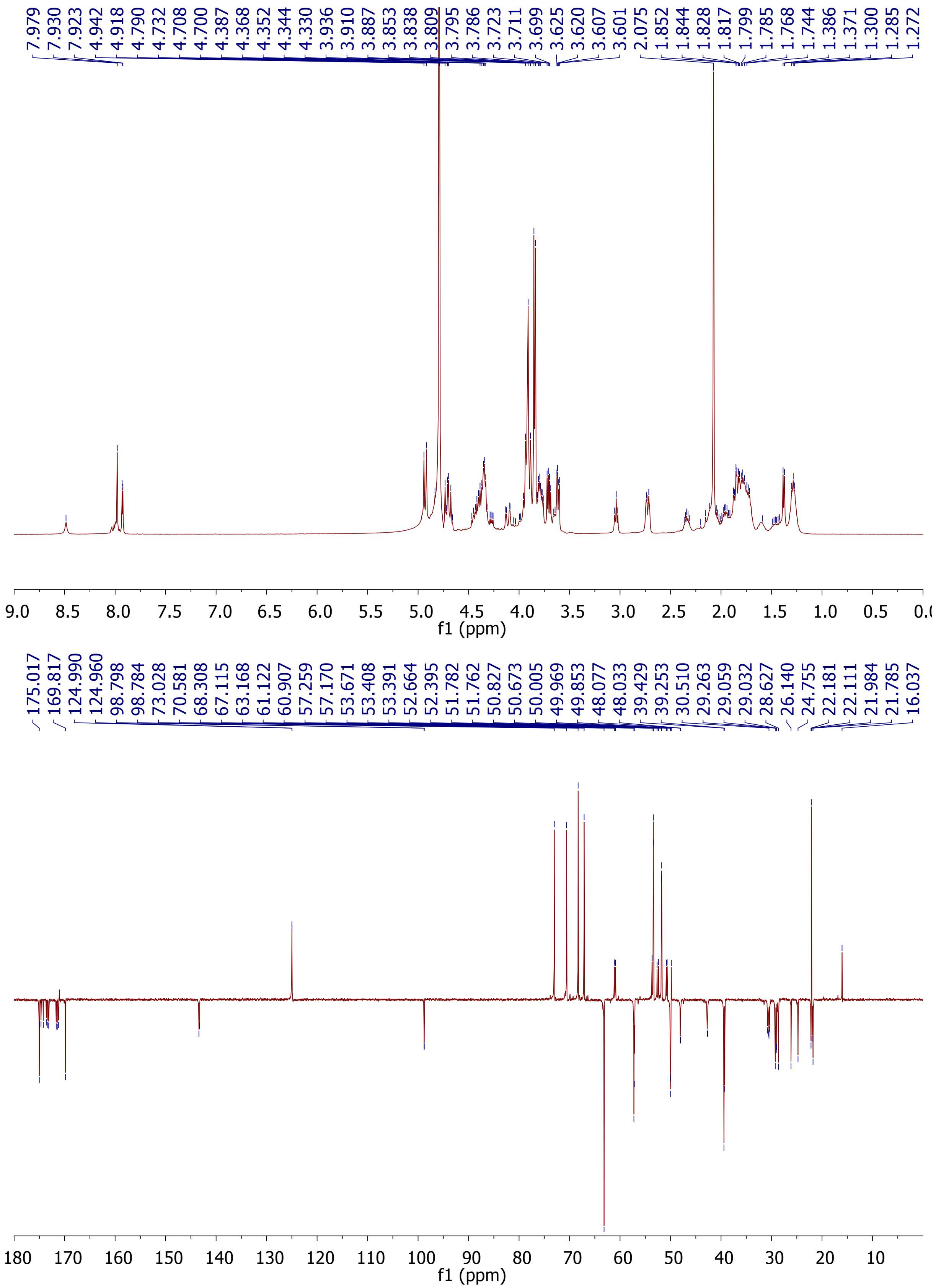

Figure S19. ^{1}H (top) and ^{13}C NMR (bottom) spectra of compound **7** in D_2O at 500 and 125 MHz, respectively.

Azido-functionalized sialylated tetravalent glycocluster (8)

To a solution of **7** (50 mg, 19 µmol, 1 eq.) in dry DMF (500 µL) were added diisopropylethylamine (9.9 µL, 57 µmol, 3 eq.) and *N*-succinimidyl azidoacetate (5.6 mg, 28.5 µmol, 1.5 eq.). The mixture was stirred at r.t. for 1 h after which UPLC analysis showed complete conversion. H_2O (3 mL) was then added to the mixture which was purified by semipreparative RP-HPLC (5-40% solvent B in 15 min) to afford compound **8** (38.0 mg, 14.3 µmol, 75%) as a white fluffy solid after lyophilization. HRMS (ESI$^+$-TOF): *m/z* calcd for $C_{109}H_{170}N_{30}NaO_{47}$ [M+Na]$^+$ 2674.1732; found 2674.1736; RP-HPLC: R_t = 7.93 min. (C18, λ = 214 nm, 5-40% solvent B in 15 min).

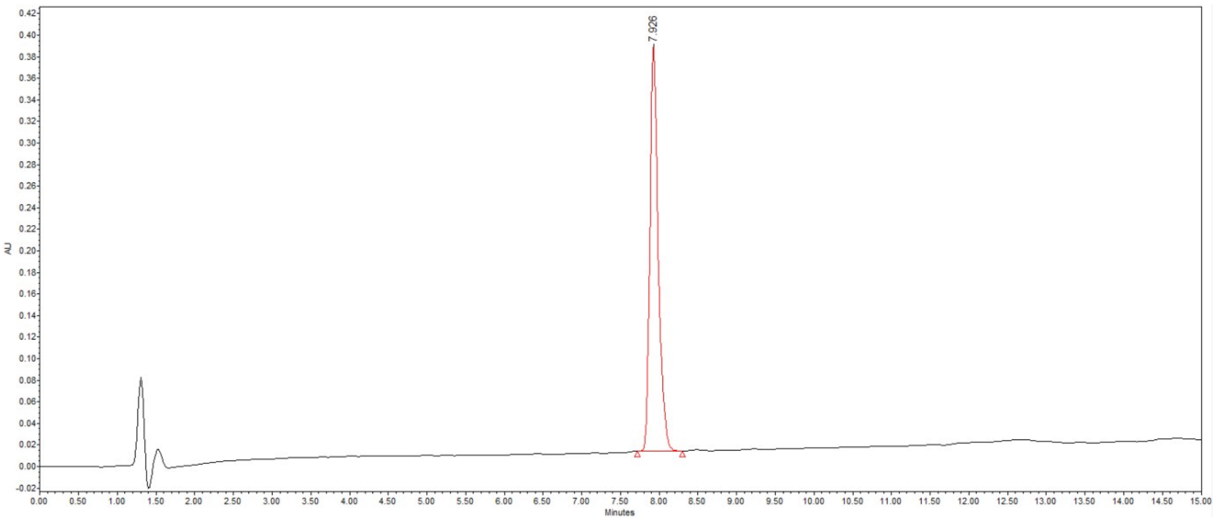

Figure S20. Analytical HPLC spectrum of compound **8**.

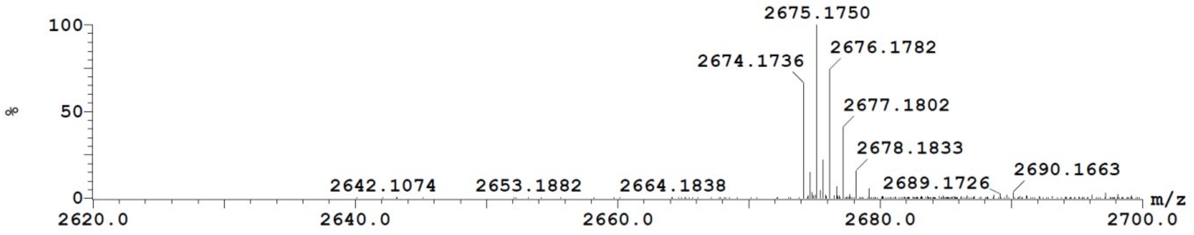

Mass	PPM	i-FIT (norm)	Formula	Calc. Mass
2674.1736	0.1	4.2	C109 H170 N30 O47 Na	2674.1732

Figure S21. HRMS spectrum of compound **8**.

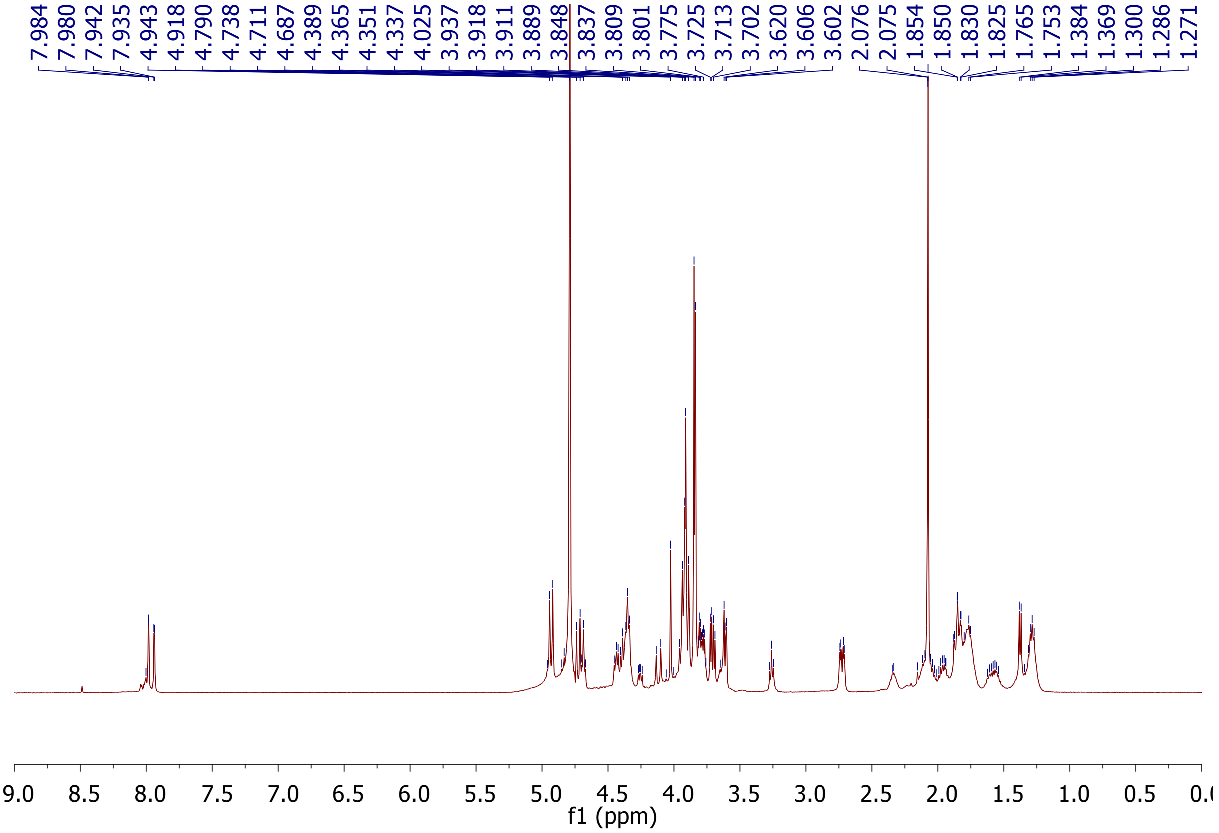

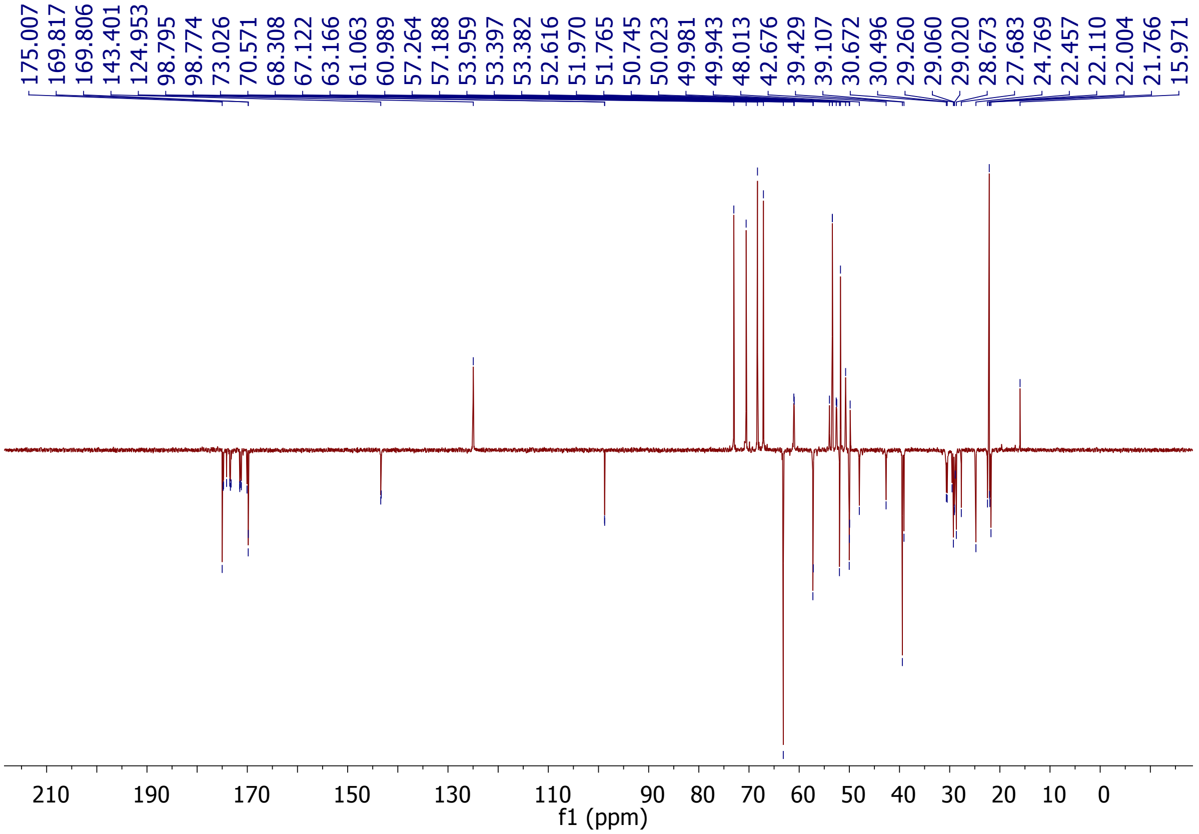

Figure S22. ^{1}H (top) and ^{13}C NMR (bottom) spectra of compound **8** in D_2O at 500 and 125 MHz, respectively.

Octavalent heteroglycocluster (2)

A solution of $CuSO_4 \cdot 5H_2O$ (0.2 mg, 0.7 µmol, 0.1 eq.), THPTA (0.6 mg, 1.4 µmol, 0.2 eq.) and sodium ascorbate (0.7 mg, 3.5 µmol, 0.5 eq.) in PBS buffer (500 µL, pH 7.5) was added to a solution of glycocluster **6** (18.7 mg, 7 µmol, 1 eq.) and glycocluster **8** (18.7 mg, 7 µmol, 1 eq.) in PBS buffer (500 µL, pH 7.5). The mixture was degassed under argon and stirred at room temperature for 1 h after which UPLC analysis showed complete coupling. LiOH (1.7 mg, 70 µmol, 10 eq.) was then added and the mixture stirred at r.t. for 1 h more. Chelex resin was added to the reaction mixture which was then purified by semipreparative RP-HPLC (5-40% solvent B in 15 min) to afford compound **2** (23.0 mg, 5.0 µmol, 71%) as a white fluffy solid after lyophilization. HRMS (ESI$^+$-TOF): *m/z* calcd for $C_{193}H_{299}N_{53}O_{78}$ [M+H]$^+$ 4607.1060; found 4607.1015; RP-HPLC: R_t = 6.53 min (C18, λ = 214 nm, 5-40% solvent B in 15 min).

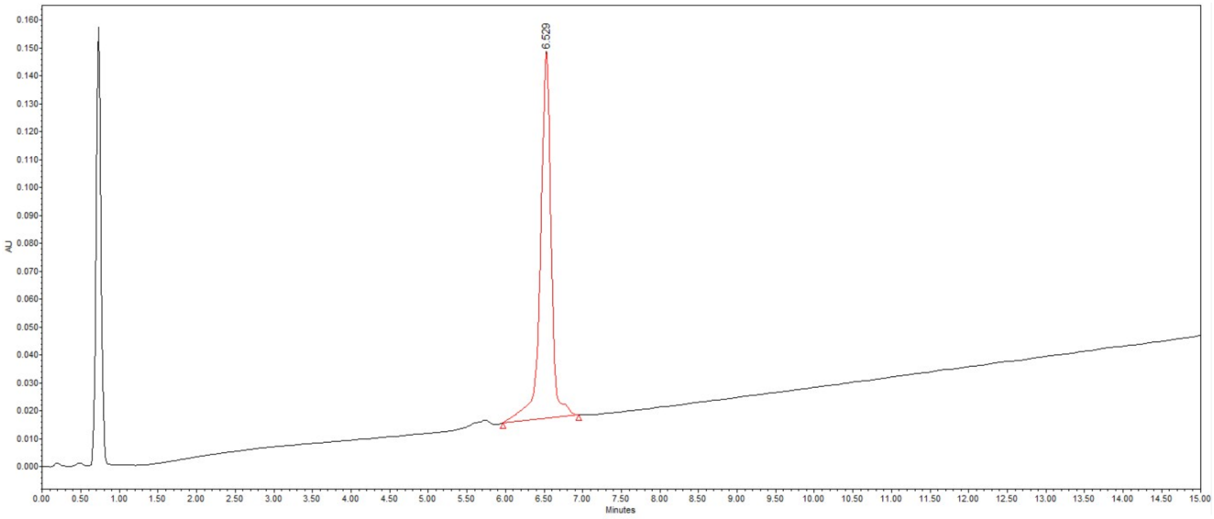

Figure S23. Analytical HPLC spectrum of compound **2**.

**M target**	**4607.1060**
**M**	**4607.1015**
**erreur en PPM**	**0.98**

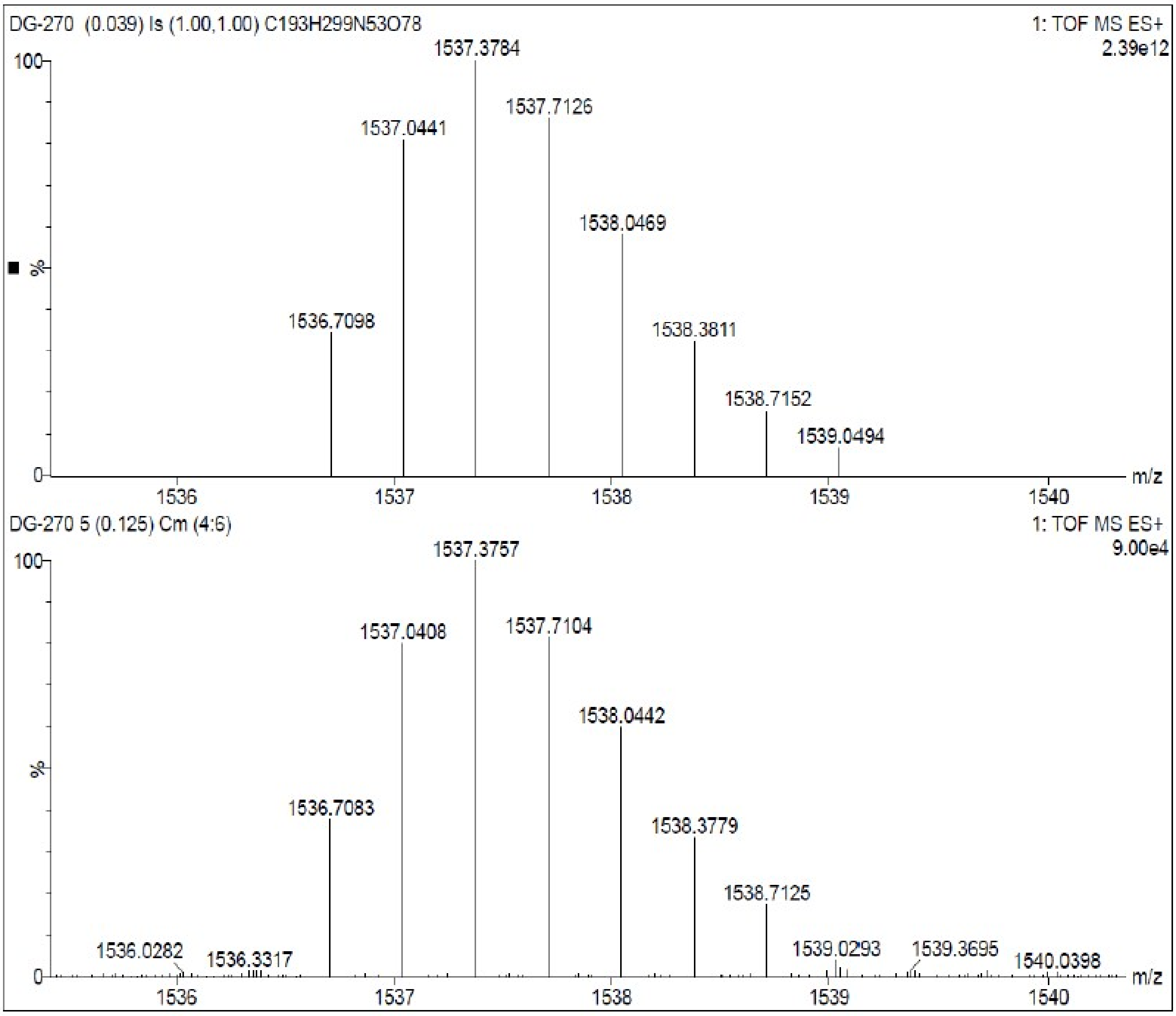

Figure S24. HRMS calculated (top) and measured (bottom) spectra of compound **2**.

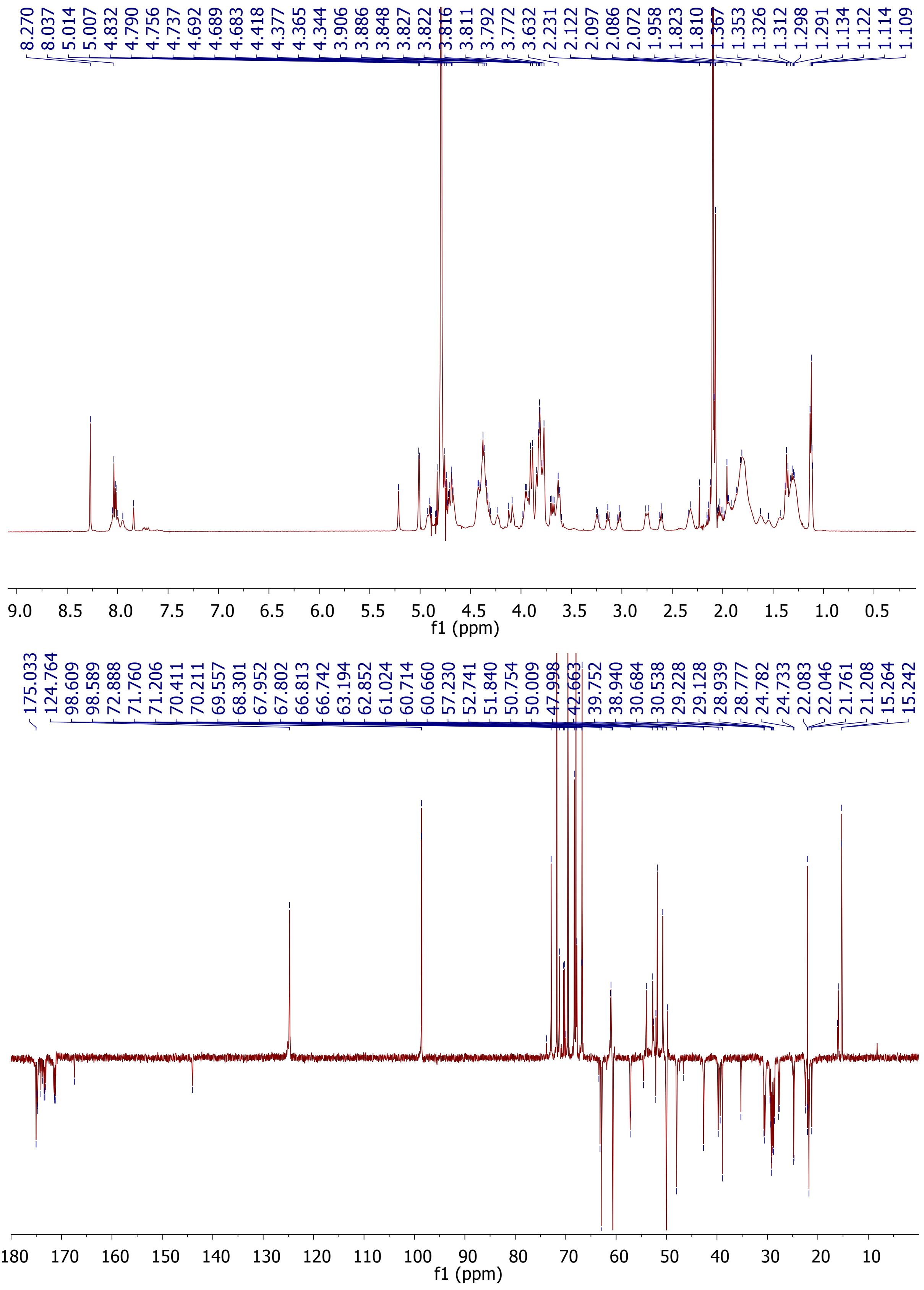

Figure S25. ^{1}H (top) and ^{13}C NMR (bottom) spectra of compound **2** in D_2O at 500 and 125 MHz, respectively.
